# Pre-contact Agave domesticates – living legacy plants in Arizona’s landscape

**DOI:** 10.1093/aob/mcad113

**Published:** 2023-10-10

**Authors:** Wendy C Hodgson, E Jane Rosenthal, Andrew M Salywon

**Affiliations:** Desert Botanical Garden, 1201 N. Galvin Parkway, Phoenix, AZ 85008, USA; Desert Botanical Garden, 1201 N. Galvin Parkway, Phoenix, AZ 85008, USA; Desert Botanical Garden, 1201 N. Galvin Parkway, Phoenix, AZ 85008, USA

**Keywords:** Agave, domesticates, pre-Columbian, Aridamerica, Arizona, borderlands, dry-land farming

## Abstract

**Background and Scope:**

Agaves played a central role as multi-use plants providing food, fibre and beverage to pre-contact and historical Mesoamerican cultures. However, their importance to Indigenous Peoples in the Southwest USA and northern Mexico, where they occur because of adaptations such as CAM photosynthesis, is less well known. Archaeological research indicates the Hohokam and other pre-contact Southwestern agrarian people increased agricultural potential in this region by engineering riverine terraces and bajadas for agave dry farming. Agricultural features such as terraces and rock piles were especially characteristic of post-1000 CE with the increase of dense, aggregated populations. We present an overview of six pre-contact agave domesticates (PCADs) the Hohokam and other cultures cultivated, and their ecological and cultural attributes. These PCADs are *Agave murpheyi*, *A. delamateri*, *A. phillipsiana*, *A. sanpedroensis*, *A. verdensis* and *A. yavapaiensis.*

**Conclusion:**

Pre-contact agriculturists cultivated at least six once cryptic domesticated agave species in the modern Arizona landscape associated with pre-contact agricultural features, such as rock structures. Because of the longevity and primarily asexual reproduction of these agaves, relict clones have persisted to the present day, providing an opportunity to study pre-contact nutrition, trade, migration and agricultural practices. Taxonomic data imply that pre-contact farmers selected desirable attributes, initiating domestication processes that resulted in discrete lineages. These agaves are morphologically and genetically distinct from Southwest US and northern Mexico wild agaves and Mesoamerican wild and domesticated species. Additionally, the remnant clones present a rare opportunity to examine domesticates virtually unchanged since they were last cultivated prehistorically. These discoveries underline the need to view landscapes and some plant species from a cultural, rather than ‘natural’, perspective and discern potential cryptic species veiled by traditional taxonomic treatments. Protecting and understanding the distribution, and ecological and cultural roles of these plants require interdisciplinary collaboration between botanists, archaeologists, federal agencies and Indigenous Peoples.

## INTRODUCTION

The genus *Agave sensu lato* (including *Manfreda*, *Polianthes* and *Prochnyanthes*) comprises over 250 species in the American subfamily Agavoideae, Asparagaceae (Jimenez-Barron *et al.*, 2020). Agave’s greatest diversity is in central Mexico, from where it spread and diversified into the southwestern USA, Central America, the Caribbean and northern South America (Bogler *et al*., 2006; Good-Avila *et al*., 2006; Jimenez-Barron *et al*., 2020). Agaves grow from sea level to 2450 m (8000 ft) and thrive on well-drained soils, particularly those derived from limestone or igneous rocks (Gentry, 1972). The genus is also prominent in arid and semi-arid regions with adaptations, including Crassulacean acid metabolism (CAM), that allow the plants to survive extreme heat, cold and drought (Nobel, 2010; Stewart, 2015; Heyduk *et al*., 2016).

Agaves have short, thick stems as small as 10 cm (4 inches) or as large as 2.5 m (8 ft) tall, their size generally decreasing the more northerly the latitude (Gentry, 1972). Agaves are rosette-forming succulents to non-succulents, with fibrous and variously shaped spine-tipped leaves that often have marginal teeth or fibres. Agaves are usually monocarpic perennials taking 7 to more than 40 years to mature before producing a flowering stalk from the apical meristem and usually expiring. Agaves reproduce by sexual and/or asexual, or vegetative, reproduction. Vegetative reproduction is via three cloning mechanisms: (1) ramets or *chupones* (suckers) that are produced from the axillary leaf meristem in the lower main stem at the base of the usually adult plant; (2) *hijuelos* or pups derived from axillary (secondary) bract meristems in rhizomes that are not from the main stem (pups emerge at a distance from the mother plant); and (3) smaller plantlets called bulbils or *bulbilos* derived mainly from axillary buds on the sides of pedicels when flowers abscise (Szarek *et al*., 1996; Arizaga and Ezcurra, 2002). The production of ramets is the most common mode of asexual reproduction. Numerous agaves employ both sexual and vegetative means of reproduction. Vegetative reproduction creates rosettes that are genetically identical or nearly identical.

Agaves have been of great economic and social importance in Mesoamerica (central and southern Mexico and Central America) and arid America (Colunga-GarcíaMarín and May-Pat, 1993) for at least 9000 years (Callen, 1965; Smith, 1965; Zizumbo-Villarreal and Colunga-GarcíaMarín, 2010) and in the American Southwest since ca. 5000 BCE (Callen, 1965). Before corn was cultivated, agaves were one of the main carbohydrate sources (Zizumbo-Villarreal *et al*., 2009). The preferred food preparation method was pit baking the emerging floral peduncles (‘quiotes’) and particularly the carbohydrate-rich ‘heads, *cabezas*, or *piñas*’ with attached leaf bases, a practice virtually unchanged since 9000 BCE (Callen, 1965; Colunga-GarcíaMarín and Zizumbo-Villarreal, 2006); Supplementary Data Fig. S1 depicts agave hearts and pit baking. Especially important in arid regions where water is limited, long-term moist heating by pit baking breaks down agaves’ complex fructans into an easily digested, sweet oligofructose (Schneeman, 1999; Pérez-López and Simpson, 2020).

Agave cultivation may have begun 6000–5000 BP in Mesoamerica (Zizumbo-Villarreal *et al*., 2009), achieving great economic and social significance (Colunga-GarcíaMarín and May-Pat, 1993) providing food, fibre, beverage and ritual paraphernalia (Callen, 1965; Gentry, 1982; Bruman, 2000; Colunga-GarcíaMarín and Zizumbo-Villarreal, 2006; Zizumbo-Villarreal *et al*., 2009; Zizumbo-Villarreal and Colunga-GarcíaMarín, 2010). However, in the American Southwest and northwestern Mexico early explorers and ethnographers did not observe or mention agaves being cultivated (Fish and Fish, 2014). Rather, they recorded only wild agaves that Southwest native peoples collected and/or traded for food, fibre, and making paper, soap, shampoos, needles, medicines, armed fences and fermented beverages, as well as being used in construction and ceremonial activities, and as ornamentals (Castetter *et al.*, 1938; Callen, 1965; Gentry, 1982; Hodgson, 2001*a*). Given agave’s importance it was reasonable to suppose that pre-contact Indigenous Peoples once cultivated agaves in the Southwest, as its presence was too ubiquitous and its use too extensive to sustain people’s needs solely by gathering (Bohrer, 1991). Southwestern agaves were, and continue to be, excellent candidates for arid land cultivation, representing a relatively reliable and stable resource (Nabhan *et al*., 2020; Eguiarte *et al*., 2021). However, questions remain regarding its importance to the region’s early Indigenous Peoples.

## ARCHAEOLOGICAL EVIDENCE FOR LARGE-SCALE AGAVE CULTIVATION IN THE SOUTHWEST

Descendants of local Archaic hunter-gatherers started growing maize within Arizona’s major river valleys ca. 2100 BCE (Andrews and Bostwick, 1997; Diehl, 2009; Nials *et al.*, 2011) and 500 years later began constructing irrigation canals watering their fields. Since ca. CE 450–600, a distinct cultural tradition archaeologists called Hohokam, or what present-day O’odham descendants called Huhugam, and Sinagua expanded irrigation systems in southern and central Arizona, respectively (Andrews and Bostwick, 1997), while harvesting crops of Mesoamerican origin – maize, beans, squashes, bottle gourds, amaranth and cotton (Gasser and Kwiatkowski, 1991; Fish, 2004; Fritz *et al*., 2009). Large villages, with public features such as the ceremonial ball court, platform mounds, markets, reservoirs and multi-storey great houses, occurred regularly along Arizona rivers (Abbott *et al*., 2007; Clark *et al.*, 2012). By CE 1300, the Hohokam population approached 40 000 people (Hill *et al*., 2004), one of the largest concentrations in the pre-contact American Southwest.

Archaeological cultural resource research from 1980 to the present has revealed evidence for intensive pre-contact agave agriculture in Arizona. The first extensive description of agave cultivation identified ridged, gridded, rock piled constructs and roasting pits containing carbonized macro-botanical agave remains on Santa Cruz River pediments and bajadas near Marana, north of Tucson (Fish *et al*., 1985, 1992). Contour terraces, rock alignments and check dams (rock alignments that cross drainages) often occurred with rock piles in fields ranging from a few rock-pile clusters to many hectares (Fish *et al*., 1992) and were associated with Hohokam towns and villages located along the riverbanks. During the following 20 years, archaeologists identified similar rock constructs near villages containing agave remains along the Salt, Gila (Crown, 1984), Verde (Whittlesey *et al*., 1997) and New River (Rankin and Katzer, 1989) rivers, and at Lake Roosevelt at the confluence of Salt River and Tonto Creek (Spielmann, 1998).

**Fig. 1.**
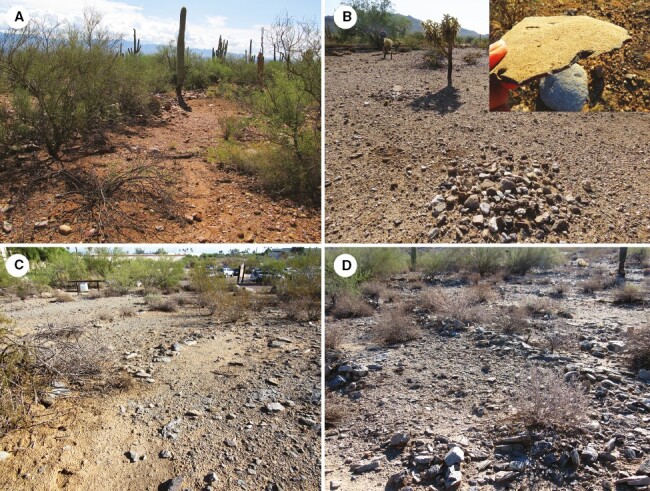
Hohokam rock mulch features. (A) Terraces (note vegetation along rock borders); (B) rock piles (inset: agave processing knife) – Queen Creek; (C, D) agave terraces (Phoenix).

The association of agaves and rock piles started before the early Classic Period as archaeologists dated a few small complexes of rock piles, linear stone features, and roasting pits prior to 1000 CE (Fish *et al.*, 1985). Rock pile technology grew significantly post-1000 CE when greatly expanded fields distant from villages coincided with dense, aggregated populations (Fish *et al*., 1992). Rock piles and alignments (Fig. 1) capture and retain moisture and nutrients, increase rain infiltration, slow evaporation and surface water flows, aerate soil, increase soil organic matter, provide insulation to roots, and deter gopher predation (Fish *et al*., 1985; Homburg *et al*., 2011; Hodgson, 2013; Hodgson *et al.*, 2018; Ortiz-Cano *et al.*, 2020).

Artefacts, features and macro-botanical archaeological remains included agave fragments and agave-processing tools such as core scrapers/pulping planes and agave tabular kneives (Fig. 1B inset), flaked and ground stone processing tools and roasting pits, evidence to suggest that agaves were widely cultivated in arid-land constructed fields (Fish *et al.*, 1985, 1992; Bohrer, 1987). Early harvesters used tabular knives to sever the green leaves, and the core scrapers and pulping planes to scrape and expose fibres and strip off marginal spines before leaf transport (Fish *et al*., 1992). Archaeologists suggested that growing agaves near settlements concentrated a normally dispersed resource making harvesting and transport easier. Given the apparent population increase from 700 CE onward, researchers proposed that agaves were a late winter and spring food supplement and/or provided an increase in fibre availability (Crown, 1984). Mound structures, roasting pits and macro-botanical remains, as well as pottery vessels indicated consumption during feasting or rituals when possibly a beverage was prepared (Lindauer, 1996; Spielmann, 1998; Russell *et al*., 2011).

## PRE-CONTACT AGAVE DOMESTICATES IN THE SOUTHWEST – THE PCADS

Archaeologists found no agave living in Marana sites or along the Salt and Gila Rivers. The closest documented wild species, *Agave palmeri* Engelm., *A. chrysantha* Peebles and *A. simplex* Salywon & W.C. Hodgson, occur ~25, 28 and 35 km, respectively, from the Marana fields. *Agave parryi* Engelm. var. *parryi* occurs ~30 km from these rock pile sites at higher elevations. Macro-botanical archaeological specimens from sites were insufficient for species-specific identification. Researchers speculated that the species cultivated may have been wild agaves native to Arizona or perhaps Mexican cultigen/domesticates obtained by trade (Fish *et al*., 1985). Indeed, pre-contact cultures cultivated wild agaves (Minnis and Plog 1976; Parker *et al*., 2010, 2014; Spielmann *et al*., 2011) including an undescribed species in central Arizona (see Supplementary Data Figs S2–S5 for images of these wild species).

**Fig. 2.**
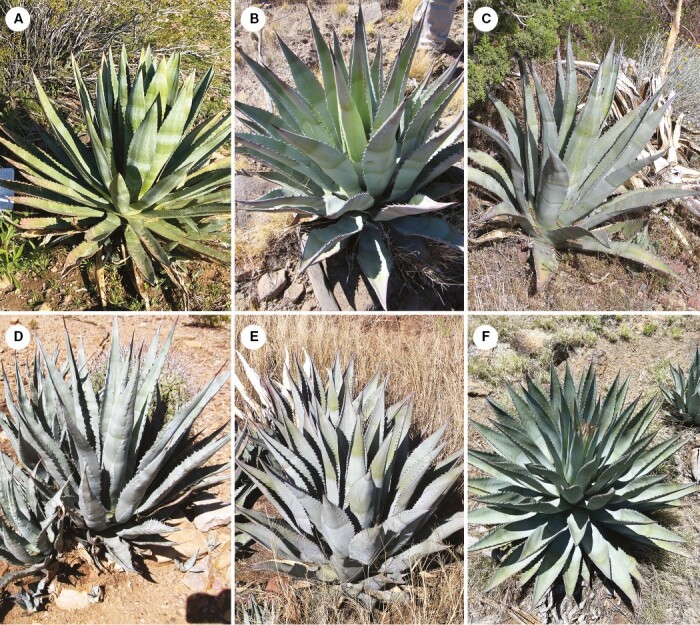
The pre-contact agave domesticates (PCADs). (A) *Agave murpheyi* (north of Phoenix); (B) *Agave delamateri* (Tonto Basin); (C) *Agave phillipsiana* (Sedona); (D) *Agave sanpedroensis* (San Manuel area); (E) *Agave verdensis* (Verde Valley); (F) *Agave yavapaiensis* (Verde Valley).

**Fig. 3.**
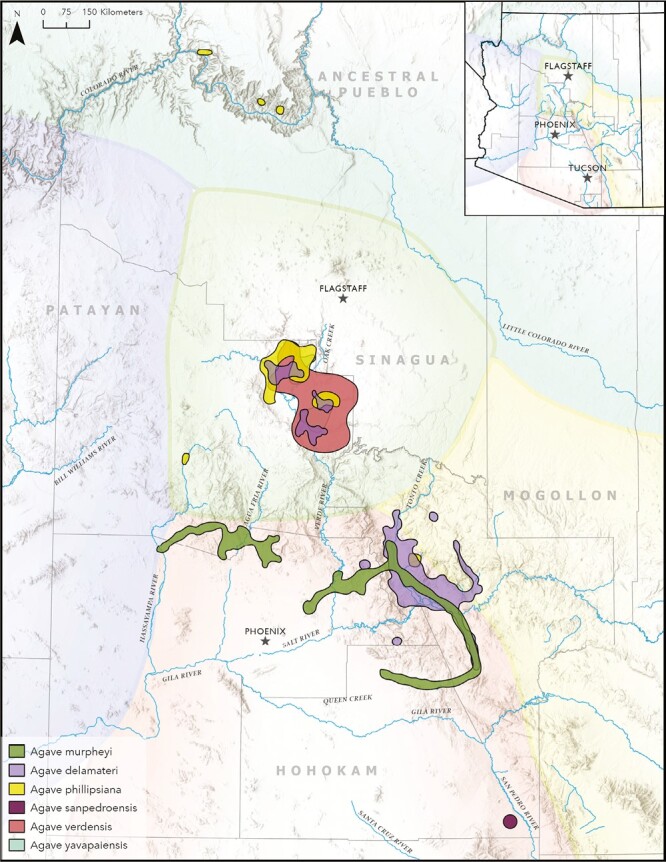
Approximate areas of documented PCADs and the cultures who farmed them prior to the 1350s (based on an original map by Catherine Gilman, courtesy of Archaeology Southwest). These areas represent a small portion of the total amount of land on which pre-contact cultures cultivated the PCADs. Several PCADs occur within the Verde Valley, often growing together.

**Fig. 4.**
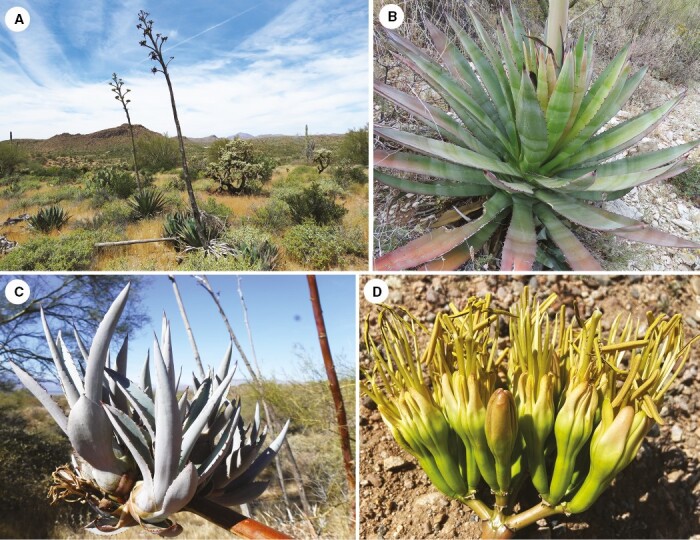
*Agave murpheyi.* (A) Habit showing narrow inflorescence (east of Phoenix); (B) rosette and leaves with short terminal spine and marginal teeth; (C) inflorescence with bulbils; (D) greenish-yellow flowers with maroon flush.

**Fig. 5.**
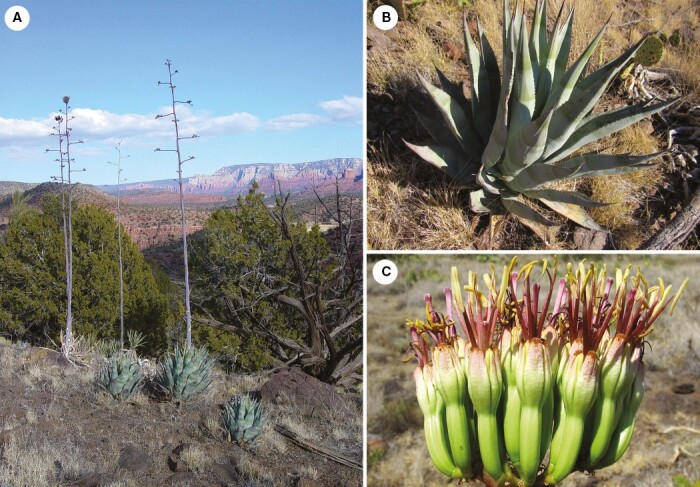
*Agave delamateri.* (A) Clone with characteristic tall inflorescence and long, widely separated lateral branches (Sedona); (B) close-up of rosette showing large grey-green leaves flushed with maroon; (C) characteristic large, thick maroon-light green flowers.

**Fig. 6.**
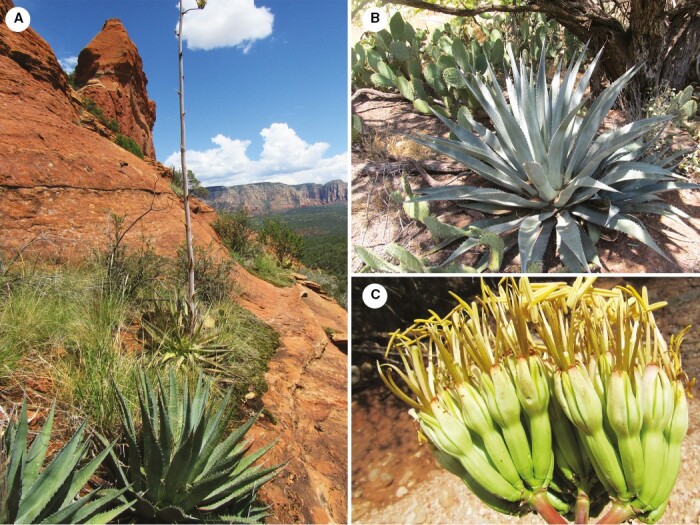
*Agave phillipsiana.* (A) Clone showing dark green leaves (Sedona); (B) characteristic large, open rosette with glaucous green to dark green leaves, the marginal teeth variable in orientation (Grand Canyon); (C) large, thick, light green-cream flowers, the tepals, filaments and style flushed with maroon.

**Fig. 7.**
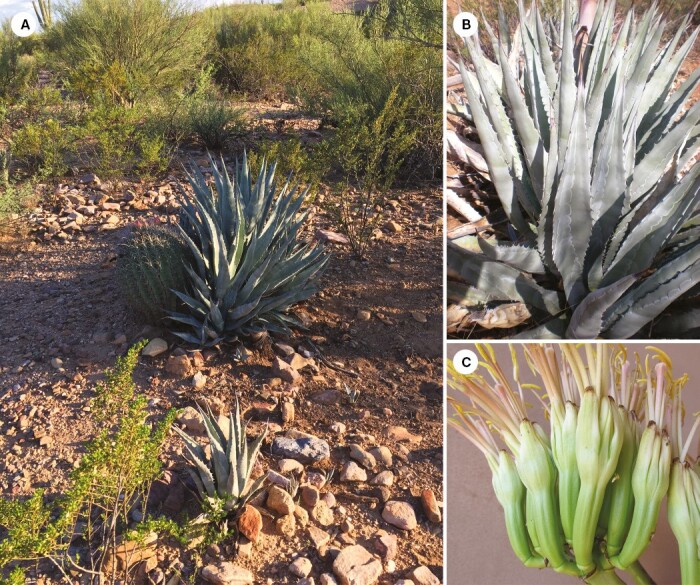
*Agave sanpedroensis.* (A) Plants within terraces and large rock piles (San Manuel); (B) rosette with characteristic conspicuous cross-banding and white imprinting on leaves; (C) close-up of large, thick, light green-cream flowers flushed with maroon.

**Fig. 8.**
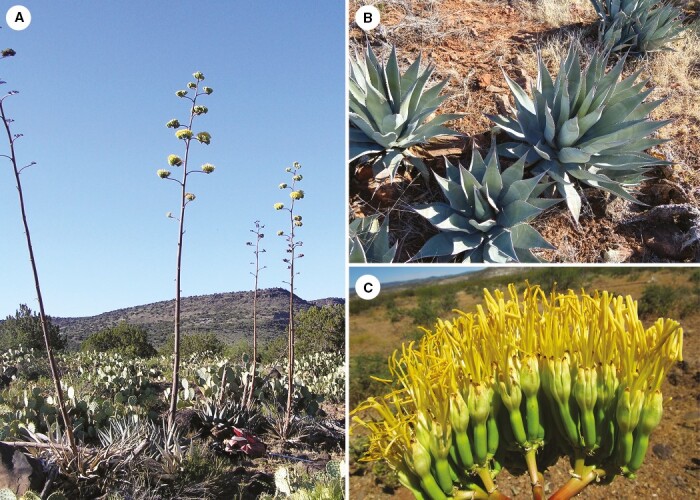
*Agave verdensis.* (A) Clones showing narrow inflorescences and synchrony of flowering typical of PCADs (Verde Valley); (B) close-up of compact rosettes, and the light glaucous-grey-green widely oblanceolate leaves with many small deflexed teeth; (C) small, light green-cream flowers with thick, clasping, firm tepals and deep tube.

**Fig. 9.**
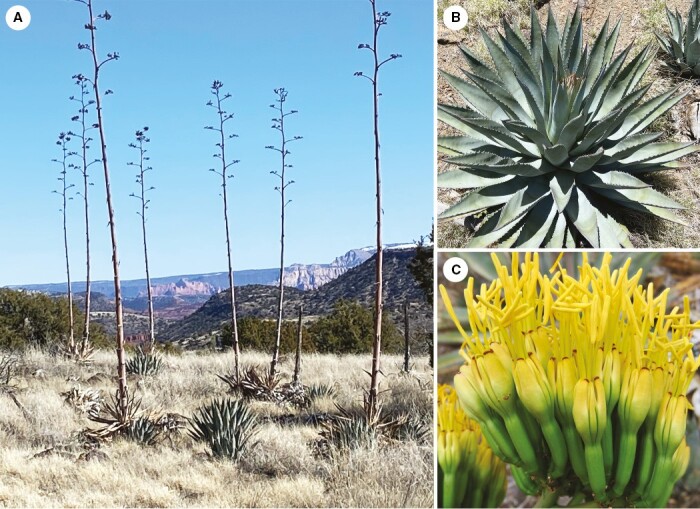
*Agave yavapaiensis.* (A) Clones atop ridge overlooking permanent water, showing few fruits produced on uppermost branches of flower stalk (Verde Valley); (B) close up of compact rosette and grey-green linear-lanceolate leaves with numerous, closely spaced marginal teeth; (C) small flowers with light green ovaries and firm, clasping yellow tepals.

Concurrent with the archaeological research, Desert Botanical Garden staff have undertaken surveys throughout much of Arizona, including around known Hohokam, Sinagua and Ancestral Pueblo fields and villages. They have rediscovered and described five of the six known agave species representing remnant populations of pre-contact domesticated agaves (herein called PCADs) that researchers have previously not found or overlooked (Hodgson and Slauson, 1995; Hodgson, 2001*a*, *b*; Hodgson and Salywon, 2013; Hodgson *et al*., 2018). The PCADs are *A. murpheyi* F. Gibson, *A. delamateri* W.C. Hodgson & Slauson, *A. phillipsiana* W.C. Hodgson, *A. sanpedroensis* W.C. Hodgson & Salywon, *A. verdensis* W.C. Hodgson & Salywon and *A. yavapaiensis* W.C. Hodgson & Salywon (Figs 2–9) (see Supplementary Data Tables S1–S6 for vouchered specimen accessions).

Our data suggest the Hohokam grew at least four PCADs, the plants occurring near major archaeological sites in the New River and Tortolita Mountains foothills, along Queen Creek, Agua Fria and Hassayampa rivers, Salt River/Roosevelt Lake areas north and east of Phoenix, and along the San Pedro River, east of Tucson. Along the upper Verde River four PCADs occur, providing support that the Sinagua grew at least four agaves, while Ancestral Pueblo and perhaps Patayan and Mogollon grew at least one PCAD, which still grows in the Grand Canyon and southwest of Prescott, respectively (Fig. 3; Tables 1 and 2) (see Supplementary Data Fig. S6 for individual maps of each PCAD).

**Table 1.** Cultures who farmed the PCADs, chronology, location, biome/habitat and agaves that provided food.

Archaeological culture (post-Early Ceramic, 500–1450 CE)	Chronology	Location in Arizona	Biome/habitat	Wild/cultivated *Agave* providing food (excluding trade; present distributions)
Mogollon	300 BCE – 1100 CE	Eastern and Central	Desert grassland, Interior Chaparral, oak–juniper–pine woodland	*A. parryi* (subsp*. parryi*, *huachucensis*), *A. chrysantha*, *A. palmeri*, *A. neomexicana*, *A. gracilipes*, *A. lechuguilla*/*A. delamateri* (?), *A. phillipsiana* (?)
Ancestral Pueblo (Anasazi)	100–1600 CE	Northern	Great Basin Desert, Desert Grassland, oak–juniper–pine woodland	*A. parryi*, *A. utahensis* (subsp. *utahensis*, *kaibabensis*)/*A. phillipsiana*
Hohokam (Huhugam)	500–1450 CE	Central and Southern	Sonoran Desert, Chihuahuan Desert Desert Grassland, Interior Chaparral, oak–juniper–pine woodland	*A. chrysantha*, *A. simplex*, *A. parryi* var*. parryi*, *A. palmeri*, *A. mckelveyana*, *A.* sp. nov./*A. murpheyi*, *A. delamateri*, *A. sanpedroensis*, *A. phillipsiana*
Sinagua	600–1425 CE	Central and Northern	Desert Grassland, Interior Chaparral, oak–juniper–pine woodland	*A. chrysantha*, *A.* sp. nov., *A. parryi* (vars. *couesii*, *parryi*)*/A. delamateri*, *A. phillipsiana*, *A. verdensis*, *A. yavapaiensis*
Patayan	700–1500 CE	Western, Central and Southern	Mohave Desert, Sonoran Desert, Desert Grassland, Interior chaparral, oak–juniper–pine woodland	*A. parryi* var*. couesii*, *A. mckelveyana*, *A. simplex*, *A. chrysantha/Agave phillipsiana* (?)

**Table 2.** Characteristics of the PCADs (adapted from Hodgson, 2013^1^)

		*A. murpheyi*	*A. sanpedro*	*A. delamateri*	*A. phillipsiana*	*A. verdensis*	*A. yavapaiensis*
Distribution (all in AZ)		South-central	Southeast	Central, north-central	Central, north, north-central	North-central	North-central
Elevation (m)		540–900	914–1117	700–1500	700–1650	1000–1500	1000–1700
Biome/habitat		Sonoran Desert	Sonoran Desert	Sonoran Desert, Desert Grassland, Interior Chaparral, oak–juniper–pine woodland	Sonoran Desert, Desert Grassland Interior Chaparral, oak–juniper–pine woodland	Interior Chaparral, oak–juniper–pine woodland	Interior Chaparral, oak–juniper–pine woodland
Number of clones/populations documented		89	16	180	92	133	43
Cultures		Hohokam	Hohokam	Hohokam Sinagua Mogollon	Ancestral Pueblo Sinagua Hohokam Mogollon?	Sinagua	Sinagua
Rosette height (m)		0.6–1.2	0.5–0.7	0.6–1	0.75–1	0.5–0.6	0.5–0.6
Heart size*		Medium	Medium	Medium	Medium	Medium	Large
Leaves							
	Leaf length (cm)*	50–80	44–59	50–73	76–78	28–47	33–50
	Easily cut*	Yes	Yes	Yes	Yes	Yes	Yes
	Teeth size*	Small	Small	Small	Vary	Small	Small
	Teeth deflexed or straight*	Yes	Yes	Yes	Vary	Yes	Yes
Reproduction							
	Sexual	Rarely	No	No	No	Limited	Limited
	Asexual						
	Chupones*	Yes	Yes	Yes	Yes	Yes	Yes
	Hijuelos*	Yes	?	Yes	Yes (?)	Yes (?)	Yes
	Bulbilos* (un-damaged stalk)	Yes	No	No	No	No	No
Flowering							
	Time*	Mar–Jun; Aug–Sept	Jul–Aug	June–Aug	(May–) June–Sept	Jun–Jul	Jun–Jul
	Synchronous*	Yes	Yes	Yes	Yes	Yes	Yes
	Nectar produced	Yes	Yes	Yes	Yes	Yes	Yes
	Flower fragrance	Musky-sweet	Musky-sweet	Musky-sweet	Musky-sweet	Musky-sweet	Musky-sweet
	Flower visitors observed (birds and/or insects)	Yes	Yes	Yes	Yes	Yes	Yes
Time of harvest for food, probable beverage		Dec–March	April–May (–June?)	April–June	(March–) April–June (–July?)	April–May	April–May
Fruit		Rarely	No	No	No	Few	Few
Seed		Rarely	No	No	No	Few	Few
Possible native area		Sonora	Southern AZ	Southern AZ	Southern AZ	Central AZ	Central AZ
Chromosome no.		2*n*^2^	Polyploid	4*n*^3^	4*n*^3^	2*n*^4^	2*n*
Pollen viability		20^2^, 59, 92%	?	(14)41–86%	50–72%	(26)52–85%	81–96%
Taste* (1–5 with 5 being very sweet)		4	?	5	5	5	5

We assume that early farmers used all of the PCADs for several purposes, including food, fibre and beverage, and that they selected traits reflecting their use (indicated by an asterisk). Cloning helped fix desirable characteristics such as easily cut leaves, small, downturned teeth, production of bulbils and pups, synchrony and timing of flowering, and taste. Three of the six PCADs are polyploids; polyploidy can create a diversity of novel phenotypic traits for which people can select. Pollen viability of *Agave delamateri* plants ranged widely, from 14 and 22% in Tonto Basin to 41–86% in Verde Valley. The public compared the taste of regional wild and the PCADs baked at three agave roasts in 2007, 2008 and 2009. Tasters considered the domesticated species, especially *A. phillipsiana*, to be sweeter than the wild species. Distribution, reproductive and morphological characteristics, and flowering times are based on observations and specimens deposited at DES (see Supplementary Data Tables S1–S6 for vouchered specimen accessions); the specimens are available to view at http://swbiodiversity.org/seinet/index.php. Additional data for chromosome number and pollen viability are from W. Hodgson, *Investigations of four rare pre-Columbian cultivated agaves (Agavaceae) in central Arizona*. Final Report to US Fish & Wildlife Service (2007); and unpublished data on pollen viability analyses of agaves from Living Collection, Desert Botanical Garden (provenance data available at https://livingcollections.org/dbg/Home.aspx); copies are deposited at Desert Botanical Garden, Phoenix.

1. Hodgson (2013).

2. Pinkava and Baker (1985).

3. Reveal and Hodgson (2002).

4. Baker *et al.* (2009).

**Table 3.** List of CAM species in the Sonoran Desert (SD) used for food, beverage and medicine. Species with wider distributions in the Mohave Desert (MD) and/or Chihuahuan Desert (CD) are indicated. The wild (w), cultivated (c), pre-contact cultivated (pc) and/or pre-contact domesticate (pd) nature of the plant is indicated (for references see Hodgson, 2001*a*; Gentry, 1982; Nabhan *et al*., 2020). An asterisk (*) indicates a pre-contact domesticate outside the boundaries of the Sonoran Desert. Indigenous Peoples used additional CAM species in areas adjacent to the Sonoran Desert (e.g. *Agave mckelveyana*).

Taxon	SD	MD	CD	w	c	pc	pd
**Asparagaceae**							
*Agave aktites*	X			X	X		
*Agave americana*	X	X	X		X	X?	X?
*Agave angustifolia*	X			X	X		X?
*Agave cerulata*	X			X			
*Agave chrysantha*	X			X			
*Agave colorata*	X			X			
*Agave datylio*	X			X			
*Agave delamateri*	X						X
*Agave deserti*	X	X		X			
*Agave fortiflora*	X			X	X?	X?	X?
*Agave gigantensis*	X			X			
*Agave jaiboli*	X			X			
*Agave margaritae*	X				X?		
*Agave moranii*	X			X			
*Agave murpheyi*	X						X
*Agave palmeri*	X		X	X			
*Agave parryi*	X	X	X	X	X		
*Agave parviflora*	X			X			
*Agave pelona*	X			X			
*Agave phillipsiana*	X						X
*Agave sanpedroensis*	X						X
*Agave sebastiana*	X			X?			
*Agave shawii*	X			X			
*Agave shrevei*	X			X			
*Agave simplex*	X			X			
*Agave sobria*	X			X			
*Agave subsimplex*	X			X			
*Agave turneri*	X			X?			
*Agave utahensis*	X	X		X			
Agave verdensis*							X
*Agave vizcainoensis*	X			X?			
Agave yavapaiensis*							X
*Agave zebra*	X			X			
*Hechtia montana*	X			X			
*Yucca baccata*	X	X	X	X		X?	
*Yucca grandiflora*	X			X			
*Yucca schidigera*	X	X		X	X		
*Yucca schottii*	X			X			
*Yucca valida*	X			X			
**Cactaceae**							
*Carnegiea gigantea*	X			X			
*Cylindropuntia acanthocarpa*	X	X		X		X?	
*Cylindropuntia alcahes*	X			X			
*Cylindropuntia arbuscula*	X			X			
*Cylindropuntia bigelovii*	X	X		X			
*Cylindropuntia echinocarpa*	X	X		X			
*Cylindropuntia fulgida*	X			X	X?		
*Cylindropuntia imbricata var. spinosior*	X		X	X			
*Cylindropuntia xkelvinensis*	X			X			
*Cylindropuntia leptocaulis*	X	X	X	X			
*Cylindropuntia thurberi var. versicolor*	X		X	X		X?	
*Echinocereus engelmannii*	X	X	X	X			
*Echinocereus fasciculatus*	X	X		X			
*Echinocereus fendleri*	X		X	X			
*Echinocereus grandis*	X						
*Echinocereus nicholii*	X						
*Echinocereus scopulorum*	X						
*Escobaria vivipara*	X	X	X	X			
*Ferocactus cylindraceus*	X	X		X			
*Ferocactus emoryi*	X			X			
*Ferocactus herrarae*	X			X			
*Ferocactus tiburoensis*	X			X			
*Ferocactus viridescens*	X			X			
*Ferocactus wislizeni*	X		X	X			
*Mammillaria estebanensis*	X			X			
*Mammillaria grahamii*	X	X	X	X			
*Mammillaria tetrancistra*	X	X		X			
*Mammillaria thornberi*	X			X	X		
*Opuntia chlorotica*	X	X		X			
*Opuntia engelmannii*	X	X	X	X	X?	X?	
*Opuntia ficus-indica*	X		X		X		
*Opuntia gosseliniana*	X			X			
*Opuntia macrocentra*	X		X	X			
*Opuntia phaeacantha*	X	X	X	X			
*Pachycereus pecten-aboriginum*	X			X			
*Pachycereus pringlei*	X			X			
*Pachycereus schottii*	X			X			
*Peniocereus greggii*	X		X	X	X		
*Peniocereus striatus*	X			X			
*Stenocereus gummosus*	X			X	X?		
*Stenocereus thurberi*	X			X			
**Portulacaceae**							
*Portulaca oleracea*	X	X	X	X	X?		

These PCADs do not exist in ‘natural vegetation stands’ per se, but often grow in clonal clusters within ancient, constructed fields (Fig. 7A). Plants persist in their cultural context because they primarily reproduce asexually producing both ramets and *hijuelos*, and in the case of *A. murpheyi*, also producing *bulbilos* in the inflorescence. Only *A. verdensis* and *A. yavapaiensis* produce, albeit few, seed in the uppermost branches of the inflorescence, while *A. murpheyi* occasionally produces seed. As with other agaves that reproduce vegetatively, it appears that the production of ramets is the most common mode of vegetative reproduction in the PCADs. Although it is not known why these plants produce little if any seed (this is a topic that warrants further study), shifting from sexual reproduction to vegetative propagation is a requisite for domestication of many fruit trees (Zohary and Spiegel-Roy, 1975).

### Agave murpheyi F. Gibson ‘Hohokam Agave’

*Agave murpheyi* (Figs 2A and 4) was the first Southwest agave suggested as a possible wild agave cultivated by pre-contact agriculturists in south-central Arizona and northern Mexico (Crosswhite, 1981; Gentry, 1982; Nabhan *et al.*, 1982; Rea *et al*., 1983; Fish *et al*., 1985, 1992; Hodgson *et al*., 1989; Bohrer, 1991; Hodgson 2001*a*, *b*). It was also the first PCAD rediscovered growing amidst a major archaeological site (Hodgson *et al.*, 1989). *Agave murpheyi* occurs *in situ* between 540 and 900 m (1782–2970 ft) north of Phoenix and in the Tonto Basin, where *A. delamateri* and *A. phillipsiana* also occur (Hodgson and Slauson, 1995; Hodgson, 2001*a*, *b*, 2013; SEINet, 2022*b*). Efforts to locate potentially wild populations in southern Arizona and northern Sonora have been unsuccessful thus far. Unlike other Arizona agaves, *A. murpheyi* plants always produce many plantlets (bulbils) on the inflorescence of an undamaged flower stalk (Fig. 4C). These bulbils survive on the flower stalk for more than a year, even during droughts, and establish well after transplanting, especially within rock piles (Szarek *et al*., 1996; Adams and Adams, 1998). Such attributes enabled easy transport/trade. Evidence suggests Hohokam living north of Phoenix and Tonto Basin traded this agave (Parker *et al*., 2007). *Agave murpheyi* plants can mature from 7 to 10 years with supplemental watering (McDaniel, 1985; Adams and Adams, 1998; Fish and Fish, 2014) compared to 20–40 or more years for many other agave species. Because *A. murpheyi* initiates flowering during winter, it provided a valuable food source when fresh plant foods were often scarce and stored food reserves had dwindled. Plants flower from February to May and produce fruit and seed occasionally, relying mostly on vegetative reproduction. It is a diploid (Pinkava and Baker, 1985).

### Agave delamateri W.C. Hodgson & Slauson ‘Tonto Basin Agave’

This large-leaved agave (Figs 2B and 5), first rediscovered by Susan McKelvey in the late 1930s, occurs in central Arizona on the northern and eastern Hohokam periphery (Tonto Basin) and in Sinagua and Mogollon homelands. It occurs on alluvial terraces or ridges overlooking major drainages at elevations of 762–1676 m (2500–5500 ft) (SEINet, 2022*a*). In Tonto Basin, it grows with *A. murpheyi* and *A. phillipsiana*, and in Verde Valley grows with *A. phillipsiana*, *A. verdensis* and *A. yavapaiensis* (Hodgson, 2013) providing evidence of trade (Parker *et al*., 2007). Plants can mature in 9 years with supplementary water. Flowering occurs from July to September but the plants do not produce fruit, the flowers becoming wood-like. Plants are tetraploid (W. Hodgson, *Investigations of four rare pre-Columbian cultivated agaves in central Arizona. USFWS, Final Report*, 2007).

### Agave phillipsiana W.C. Hodgson ‘Grand Canyon Agave’

*Agave phillipsiana* (Figs 2C and 6) occurs on Ancestral Pueblo lands in the Grand Canyon, where Rose Collom first rediscovered the plant in the late 1930s. It also occurs in Sinagua, Hohokam and possibly Patayan and Mogollon lands in central Arizona. Plants grow at elevations of 700–1100 m (2500–3500 ft) in locales with distinct climates, vegetation, geology and cultures. Near Sedona, it grows with *A. delamateri*, *A. verdensis* and *A. yavapaiensis*, and in Tonto Basin grows with *A. delamateri* and *A. murpheyi* (SEINet, 2022*c*). It has the widest distribution of the six PCADs, suggesting trade among the Sinagua, Ancestral Pueblo and/or Hohokam, and possibly Patayan and Mogollon cultures. Informal taste-tests revealed it to be sweeter than other PCADs and regional wild species (W. C. Hodgson, unpubl. data). Plants flower from July to September but do not produce fruit. Plants are tetraploid (W. Hodgson, *Investigations of four rare pre-Columbian cultivated agaves in central Arizona. USFWS, Final Report*, 2007).

### Agave sanpedroensis W.C. Hodgson & Salywon ‘San Pedro Agave’

During survey work along the San Pedro River in southeastern Arizona, archaeologists recorded the first known presence of living agaves in the southern Arizona dry-farming fields among extensive rock alignments and rock piles (Clark *et al*., 2012). These agaves represented a previously undescribed, new species named *Agave sanpedroensis* (Figs 2D and 7). The few clones occur north and east of Tucson, growing in impressive rock piles and terraces (Hodgson *et al.*, 2018) at elevations of 914–1117 m (3017–3686 ft) along the San Pedro River and in the foothills of the Tortolita Mountains. The Tortolita Mountains population is only ca. 12 km from the Marana Hohokam fields as reported by Fish *et al.* (1985). Thousands of acres of rock pile fields along the San Pedro River suggest the Hohokam grew agaves on a large scale (Clark *et al*., 2012), although we know of only a dozen clones of *A. sanpedroensis* today. In cultivation in Phoenix, plants matured in 6–8 years with supplementary water; they flower in late August to September. Plants are polyploid (A. M. Salywon, unpubl. data based on flow cytometry results); we have not seen fruit.

### Agave verdensis W.C. Hodgson & Salywon

*Agave verdensis* (Figs 2E and 8) occurs in Verde Valley, between 900 and 1500 m (4000–5000 ft) in elevation (SEINet, 2022*d*). Plants occur within Sinagua sites that contain high frequencies of pre-contact agricultural features (Fish and Fish, 1984) near major settlements dating to 1130–1400 CE, and at important farming and trade activity sites dating to 1300–1400 CE (Pilles, 1981). The species grows with *A. delamateri*, *A. phillipsiana* and *A. yavapaiensis*. Localized farming and plant trading suggest that it may have been regionally significant, similar to species cultivated by the Hohokam (Gasser and Kwiatkowski, 1991). Plants flower from June to early July. It reproduces mainly by vegetative offsets, occasionally producing a few fruits with few viable seeds; it is a diploid (Baker *et al*., 2009).

### Agave yavapaiensis W.C. Hodgson & Salywon

*Agave yavapaiensis* (Figs 2F and 9) occurs in Verde Valley at 1000–1700 m (3300–5600 ft) within Sinagua sites, often occurring with *A. phillipsiana* and *A. verdensis* (SEINet, 2022*e*). Like *A. verdensis*, its farming and trade appear localized within Verde Valley, and it may have been a regionally significant plant. Plants flower from June to July. It reproduces mainly via vegetative offsets although plants occasionally produce a few fruits, with few viable seeds. It is a diploid (W. Hodgson, *Investigations of four rare pre-Columbian cultivated agaves in central Arizona. USFWS Final Report*, 2007).

## DOMESTICATION AND SIGNIFICANCE OF ARIZONA PCADS

Plant domestication is a continuous evolutionary process, driven by human selection, which fixes alleles with favourable traits for consumption and cultivation phenotypes but diminishes or eliminates their capacity to survive without human care (Harlan, 1992; Zizumbo-Villarreal and Colunga-GarcíaMarín, 2010). Pre-contact agave farmers selected for characteristics adapted to local climate and edaphic conditions, resulting in variants with discrete morphological characteristics and life cycles (Colunga-GarcíaMarín and May-Pat, 1993).

Documented PCADs appear to be domesticated species with a reduced capacity for sexual reproduction yet retaining the ability to reproduce asexually by bulbils, and/or ramets and pups. They survived unchanged as isolated, small populations or clones since the Hohokam and other pre-contact people last tended them. Because PCADs mainly reproduce via vegetative means, early farmers fixed favourable traits, including (1) cloning, (2) shorter life cycles (created with the aid of rock mulch features), (3) easily cut leaves and (4) sweeter taste (compared to wild agaves in the region). In addition, early farmers may have selected for synchrony in flowering within each species and different flowering/harvesting periods between species, the advantages described below (Hodgson, 2013; Hodgson and Salywon, 2013; Hodgson *et al*., 2018). Table 2 provides a summary of PCAD characteristics and Supplementary Data Table S7a, b compares PCADs and regional wild agave traits, including those that early farmers may have selected for, including rosette size, mode of reproduction, leaf characteristics, flowering period and taste.

Several PCADs grow together at numerous sites in Verde Valley and Tonto Basin suggesting that early farmers benefitted from synchronous flowering within each PCAD species. Although the flowering period of the PCADs is relatively short due to their flowering at the same time, synchronous flowering would help facilitate harvesting and roasting activities (Adams and Adams, 1998; Hodgson, 2013). Growing different agaves with different flowering periods extended the harvest period, while also augmenting the usually longer and more variable flowering periods of wild agaves (Hodgson, 2013). Planting different species also promoted optimal resource production adapted to local environmental or ecological conditions and uses. Supplementary Data Table S8 gives details of those agaves directly accessible to the Hohokam, Sinagua, Ancestral Pueblo and possibly Patayan cultures and Table S9 gives approximate harvesting and flowering times of regional wild and PCADs directly accessible to the Hohokam for food.

Because ancient farmers could easily transport, trade and cultivate agave bulbils and offsets, they could quickly select and perpetuate genetic variants (Gentry, 1982). With potentially hundreds or perhaps thousands of years of agricultural use there would be sufficient time to domesticate agaves from wild native ancestors (Hodgson *et al*., 2018). Because these PCADs reproduce mainly by vegetative means, there is a fortuitous opportunity to trace the genetic lineage of extant populations to their pre-contact cultivated ancestors. The plants we see today are clonal remnants of populations/species once extensively farmed with a mix of genotypes.

### Genetic variability, fixing traits, subsequent decline and putative origins

Although population genetic diversity within and between populations of *A. murpheyi* and *A. delamateri* is lower than for wild species and modern crops, these agaves have greater genetic variation than today’s extensively cultivated monocultures of *A. fourcroydes* Lem. and *A. tequilana* Web. (Parker *et al*., 2007). This diversity is similar to that found in traditional cropping where farmers cultivate several varieties, trade seeds/suckers or collect from the wild and grow landraces, maintaining diverse genotypes within fields (Parker *et al.*, 2007). In the American Southwest post-1450 CE the decline, reorganization and migration of people led to the disappearance of pre-contact management practices (Parker *et al*., 2007; Hodgson *et al.*, 2018), further eroding genetic variation (Parker *et al*., 2007). As a result, these PCADs once growing in terrace fields declined dramatically, transforming formerly cultivated landscapes to their modern ‘natural’ appearance. It is fortunate that some of these agaves persisted over the centuries, allowing us to observe, study, treasure and view the landscapes not as pristine wilderness but as a structured, indigenous-influenced, agricultural environment.

Mesoamerica is one of the global centres of plant domestication in the world (Harlan, 1971, 1992), where today people are domesticating over 200 native plant species that coexist with populations of wild relatives (Casas *et al.*, 2016), including several species of agave (Gentry, 1982; Colunga-GarcíaMarín *et al.*, 2007). Numerous studies to understand the complex interrelationships of wild to fully domesticated species under diverse agricultural practices and habitats and their effects on genetic variation continue (i.e. Casas *et al*., 1999, 2007; Miller and Schaal, 2006; Zizumbo-Villarreal *et al.*, 2009). One might assume that since most Hohokam and Sinagua irrigated crops are Mesoamerican domesticates, the agaves might have the same place of origin. Instead, these PCADs are probably part of a Southwest prehistoric and proto-historic domestication hearth (Hodgson *et al.*, 2018) that comprised at least 25 plant species (Nabhan, 1985) including Hohokam little barley (Bohrer, 1991; Adams, 2014), as well as the Ancestral Puebloan turkey, all developed independent of contemporary Mesoamerican domestics (Speller *et al.*, 2010). Morphological chloroplast sequence data suggest that *A. verdensis* and *A. yavapaiensis* of north-central Arizona have affinities with Arizona and northern Mexico agaves rather than Mesoamerican (Hodgson, 2013; Hodgson and Salywon, 2013; Hodgson *et al*., 2018). *Agave sanpedroensis* of south-central Arizona has affinities with *A. palmeri* (southern Arizona, southwestern New Mexico and northern Mexico) and *A. phillipsiana* of central and northern Arizona (Hodgson *et al*., 2018).

## CONCLUSIONS

Increasing archaeological and botanical evidence paints a picture of intensive agave cultivation in Arizona. We should not view these multipurpose agaves as minor pre-contact food and fibre plants, but as extremely valuable crops. Within the Southwest Borderlands, six and probably more PCADs have persisted in the landscape for centuries because of asexual reproduction by ramets, pups and bulbils. These PCADs and the agricultural sites where Hohokam, Sinagua, Ancestral Pueblo and possibly Patayan cultures grew them are legacies of bio-cultural, not natural, landscapes.

We propose that early farmers grew several different types of PCADs adapted to local ecological, climatological and sociological conditions for a multitude of purposes, promoting optimal resource production. Ancient DNA analysis of cordage, quids and possibly ceramic sherd analysis will potentially determine species-specific uses and time of use. With continued survey more samples will be recorded, and as molecular studies are refined, it may be possible not only to identify additional undescribed cryptic PCADs and their origins, but also document a continuum of cultivating to domestication, i.e. from the transplanting of preferred wild species through their cultivation to full domestication.

### Plants at risk

Unlike the numerous laws that protect domesticated animals (Ward, 2021), domesticated plants, including our PCADs, receive little protection. Five PCADs are listed as Sensitive Species by the US Forest Service (*A. murpheyi*, *A. delamateri*, *A. phillipsiana*, *A. verdensis* and *A. yavapaiensis*), three (*A. murpheyi*, *A. delamateri* and *A. phillipsiana*) are listed as Highly Safeguarded Native Plants under the Arizona Native Plant Law and one (*A. murpheyi*) is listed as a Sensitive Species by the Bureau of Land Management. The PCADs have contextual protection if within cultural resource sites (i.e. associated with archaeological features) on federal or Arizona lands under U.S. and Arizona Antiquities Acts, as both prohibit site disturbance or resource removal without permits. Two *A. murpheyi* sites, including one cared for by a Tohono O’odham family, received Arizona Regis-TREE Awards given by a coalition of conservation groups, gardening clubs, Native American organizations and botanical gardens for the purpose of documenting and protecting both the plants and their cultural sites (Nabhan 1992). However, the Endangered Species Act (ESA) does not provide any protection to the PCADs or any other rare plant species once cultivated and/or manipulated by people (Burgess, 1994). One can argue a culturally influenced plant that has wild populations in North America before 1492 should still qualify as a native species worthy of protection (Nabhan, 1992). Whether or not extant wild populations of the PCADs or their progenitors exist in the modern landscape is unknown. A proposal to list *A. murpheyi* as threatened under the ESA by Gary Nabhan and Hodgson in 1988 was unsuccessful, the U.S. Fish and Wildlife designating it as a candidate (C2) species due to listings of higher priority that resulted in no ESA formal protection. If clones/populations of wild *A. murpheyi* plants exist unassociated with archaeological features, its ruling as a listed species would be legitimized (Burgess, 1994). Although many PCADs occur within an archaeological context, some do not; however, to determine these plants as wild is difficult without further studies. Nor can the PCADs be included in the Red List of the International Union for the Conservation of Nature (IUCN) due to their domesticated status (IUCN, 2017), even though these culturally important arid-adapted plants have persisted for hundreds of years in their bio-cultural landscape.

Box 1. **THE ETHNOBOTANY OF CAM PLANTS IN SONORAN DESERT CULTURES**

Arid regions, including the Sonoran Desert, are key to understanding and mitigating climate crisis issues. They are laboratories where changes are already happening (Nabhan *et al.*, 2020). Unfortunately, arid and semi-arid regions, often referred by most as wasteland, are under-represented in climate studies and threatened by habitat destruction, invasive species and the effects of climate change.

The Sonoran Desert is part of Aridamerica that also includes the Mohave and Chihuahuan Deserts, and is the hottest and driest area of North America. Yet, its plant life is highly diverse with over 2500 species. It is also home to nearly 20 extant Indigenous cultural groups. Thus, the Sonoran Desert is a biologically, ecologically and culturally diverse biome.

A close relationship exists between Sonoran Desert plants and the Indigenous groups who relied on them. Both evolved adaptations to limited water availability and high temperatures. Over a fifth of the Desert’s flora provided food and beverage – with legumes, cacti and seeds with hygroscopic mucilage being especially important (Brand *et al*., 1990). Adaptations of some Sonoran Desert food plants that slow or reduce water loss in their arid environment include (1) the production of fructans (*Agave* hearts, leaves, inflorescence); (2) viscous mucilage (*Opuntia* stems, flower buds, fruits; seed coats of *Salvia* spp., *Lepidium* spp., *Plantago* spp. and *Mentzelia albicaulis*); (3) galactomannan gums (*Prosopis* spp., fruit and seeds); and (4) utilization of CAM photosynthesis (most succulents, leaves, stems). The O’odham metabolism evolved under the influence of these and other consumed desert plants (Nabhan, 2002), in an arid environment characterized by extremes of temperature and available moisture. Because many of the traditional plant foods available throughout the year were episodic in their abundance, desert peoples developed a thrifty metabolism adapted to a ‘feast or famine’ food supply. The low-glycaemic diet was especially important when food was particularly abundant (Brand *et al.*, 1990). A Western diet high in simple carbohydrates and low in fibre, in contrast to the traditional low-glycaemic, high-fibre diet, significantly increases the risk of diabetes and other metabolic syndromes for Indigenous Peoples (Brand *et al.*, 1990; Nabhan 2002; Espinosa-Andrews *et al.*, 2021).

CAM plants such as cacti and agaves withstand drought and highly variable precipitation (Nabhan *et al*., 2020). The historical Aridamerican dietary dependence on CAM plants for both nutrient-dense foods and probiotic beverages may be the highest for any world region (Leach & Sobolik, 2010; Nabhan *et al*., 2020). Seventy-six per cent of Aridamerica perennial crops, including *Opuntia*, *Stenocereus* and *Agave*, are constitutive CAM plants – more than in the Mesoamerica diet (Nabhan *et al*., 2022). Nearly a quarter of Sonoran Desert food and beverage plants are constitutive CAM species with the vast majority being succulents (Table 3). Important succulents include saguaro (*Carnegiea gigantea*), pitahayas (*Stenocereus*, three species), cardón (*Pachycereus*, two species), prickly-pears (*Opuntia*, six species), chollas (*Cylindropuntia*, ten species), agaves (*Agave*, 31 species) and fleshy-fruited yuccas (*Yucca*, five species). Additionally, purslane (*Portulaca oleracea*), a facultative CAM–C3 plant, was an important edible green (*quelite*) high in antioxidant properties and omega 3 fatty acids (Ferrari *et al*., 2020). Many other CAM plants supplemented the diet of Sonoran Desert peoples (Table 3).

CAM plants provided a reliable, stable and nutritious food/beverage and medicine irrespective of environmental vagaries and crop failures (E. Ezcurra, pers. comm. 2020). For example, several native agaves and cacti, including cardón (*Pachycereus pringlei*) and the pitahayas (*Stenocereus gummosus* and *S. thurberi*), were particularly important to Baja California cultures, as they were usually unfailing resources in periods of extended drought (Aschmann, 1959; Barco, 1980). In fact, the early Jesuits allowed the Indigenous peoples to gather the hearts, fruits and seeds of these wild plants, which saved many lives when supply ships arrived late or crops failed (Aschmann, 1959; Venegas-Burriel, 1966; Barco, 1980).

Pre-contact Sonoran Desert cultures relied heavily on CAM plants, cultivating wild species including *Opuntia engelmannii* (Pinkava, 2012), *Cylindropuntia* spp. (Fish, 1984; Bohrer, 1991), *Agave parryi* (Minnis and Plog, 1976; Parker *et al.*, 2014) and domesticated agaves (*Agave*; see text). Mesquite fruits, cactus seeds and other plant foods appear in the archaeological record as early as 5000–4000 years ago (Doolittle and Mabry, 2006; Leach and Sobolik, 2010). By cultivating several different species of agaves, prickly-pears and possibly other succulent species using dry farming strategies, Indigenous Peoples expanded agricultural potential and crop diversity.

Aridamerica arid-adapted plants can provide ideal food crop candidates. Nabhan *et al*. (2020) emphasize that investing in these plants addresses food security challenges, contributes to less fossil fuel use, more carbon drawdown, community and personal health, and fewer climate change impacts, such as habitat loss and degradation. There is extensive traditional knowledge regarding the use and cultivation of these food plants (Felger, 1975; Felger and Moser, 1985; Hodgson, 2001*a*; Nabhan *et al*., 2020). Ethnographic and palaeo-archaeological data, in addition to supporting and involving Indigenous Peoples and their traditional knowledge (including farming ways), are critical first steps in recognizing the importance of desert plants, including those utilizing CAM, and integrating them into more sustainably grown and processed healthy foods.

Since there is limited protection afforded to the PCADs for their survival, the question remains as to what other forms of protection we can provide to the PCADs and other rare culturally important species. The conservation of rare wild species involves their natural habitats and the human influences that effect those habitats (Burgess, 1994). However, the conservation of rare manipulated and domestic plants is more multi-faceted, involving complex human/plant interrelationships, requiring a diverse array of disciplines such as sociology, health, economics and ethics, as well as factors such as lifestyles and human communities (Nabhan 1985). How to protect rare culturally important species also requires addressing controversial questions as stated by Burgess (1994: 129), including ‘where do we draw the line at the edge of “natural history” where native, Indigenous People have been in contact with the plants surrounding them for hundreds or thousands of years? Do we define a natural ecosystem without *Homo sapiens* in areas where Indigenous People have clearly been a part?’ Additionally, what is a so-called natural environment if influenced by human activity decades and even centuries ago?

Understanding the relationships between Indigenous People and their threatened useful plants can aid broad-based conservation efforts on many levels (Burgess, 1994). The PCADs represent a living connection to Indigenous Peoples’ ancestors. If these and other culturally important plants are to survive, it is critical that those involved with conservation mandates alert, encourage and support tribal governments’ efforts to protect them on the local and national level ‘for the well-being of their cultures as well as for the preservation of genetic information that their ancestors may have helped to select’. (Burgess 1994: 129). This can involve the inclusion of culturally important plants as part of native plant protection ordinances for tribal lands. Providing protection to these agaves is particularly relevant today considering the increased interest and research in crop wild relatives, particularly CAM photosynthesizing plants that occupy hot, dry environments (Felger, 1979; Nabhan *et al.*, 2020). With the critical need to diversify agriculture, grow less water-dependent crops and stimulate new industries in the southwestern United States, we have the opportunity to bring these ancient agave crops back to life (Hodgson *et al.*, 2018; Nabhan *et al.*, 2022).

The PCADs provide opportunities for studying and understanding past and present cultural contexts. Understanding the origins of these species will lead to a better understanding of Southwest arid-land plant domestication. Although most pre-contact Southwestern seed crops were Mesoamerican cultigens, the PCADs represent regionally separate domestication events, their discovery adding a significant story to Southwest prehistory.

## SUPPLEMENTARY DATA

Supplementary data are available at *Annals of Botany* online and consist of the following.

Tables S1–S6. DES herbarium accession numbers of *Agave murpheyi*, *A. delamateri*, *A. phillipsiana*, *A. sanpedroensis*, *A. verdensis* and *A. yavapaiensis*, respectively. Table 7. Characteristics of Arizona cultivated agaves and regional wild agaves. Table S8. Approximate harvest time and flowering period of wild agaves and the presumed domesticated species. Table S9. Approximate harvest time and flowering times of wild agaves and the presumed domesticated species accessible to Hohokam. Figure S1. Agave as a food source, showing the hearts and roasting pit. Figure S2. *Agave palmeri*, a wild species presumably used for multiple purposes by pre- and post-contact cultures, grows ~25 km from the extensive Hohokam agave fields near Marana. Figure S3. *Agave chrysantha*, a wild species presumably used for multiple purposes by pre- and post-contact cultures, grows ~28 km from the extensive Hohokam agave fields near Marana. Figure S4. *Agave simplex*, a wild species presumably used for multiple purposes by pre- and post-contact cultures, grows ~35 km from the extensive Hohokam agave fields near Marana. Figure S5. *Agave parryi* var. *parry*, a wild species used for multiple purposes by pre- and post-contact cultures, grows ~30 km from the extensive Hohokam agave fields near Marana. Figure S6. Individual maps of the approximate areas of documented PCADs and the cultures who farmed them prior to the 1350s.

mcad113_suppl_Supplementary_Figure_S1

mcad113_suppl_Supplementary_Figure_S2

mcad113_suppl_Supplementary_Figure_S3

mcad113_suppl_Supplementary_Figure_S4

mcad113_suppl_Supplementary_Figure_S5

mcad113_suppl_Supplementary_Figure_S6

mcad113_suppl_Supplementary_Table_S1

mcad113_suppl_Supplementary_Table_S2

mcad113_suppl_Supplementary_Table_S3

mcad113_suppl_Supplementary_Table_S4

mcad113_suppl_Supplementary_Table_S5

mcad113_suppl_Supplementary_Table_S6

mcad113_suppl_Supplementary_Table_S7

mcad113_suppl_Supplementary_Table_S8

mcad113_suppl_Supplementary_Table_S9
