# Supplementary material for: Pre-contact Agave domesticates – living legacy plants in Arizona’s landscape
Source: Ann Bot. 2023 Oct 10;132(4):835–53. doi: 10.1093/aob/mcad113 (PMC10799993; doi:10.1093/aob/mcad113)
Supplement: mcad113_suppl_Supplementary_Figure_S1 [file mcad113_suppl_supplementary_figure_s1.docx]

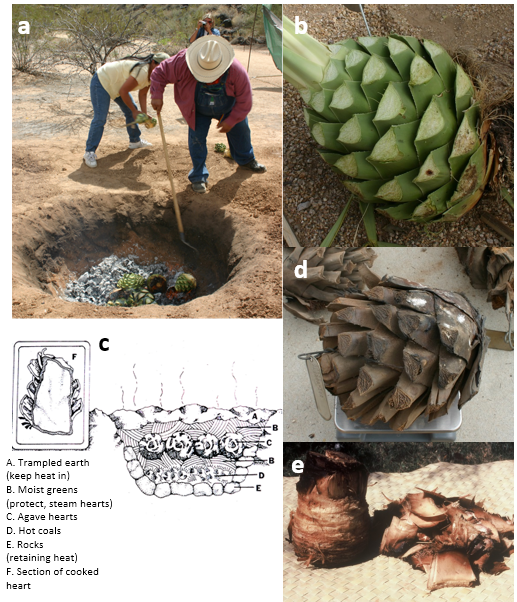


**Figure S 1:** Agave as a food source. **a**. baking the agave hearts in a pit of hot coals; **b**. generalized diagram of baking pit; **c**. prepared, unbaked agave heart; **d**. baked agave heart; **e**. baked heart and leaf bases (Valley Rock Art Center, Phoenix, April 2007; illustration cited in Hodgson 2013^1^)

**References**

1. Hodgson, W. in *Explorations in Ethnobiology: the legacy of Amadeo Rea* (Eds Quinlan, M. & Lepofsky, D*.*) 78-103 (Society of Ethnobiology, Denton, Texas, 2013).
