# Supplementary material for: Pre-contact Agave domesticates – living legacy plants in Arizona’s landscape
Source: Ann Bot. 2023 Oct 10;132(4):835–53. doi: 10.1093/aob/mcad113 (PMC10799993; doi:10.1093/aob/mcad113)
Supplement: mcad113_suppl_Supplementary_Figure_S2 [file mcad113_suppl_supplementary_figure_s2.docx]

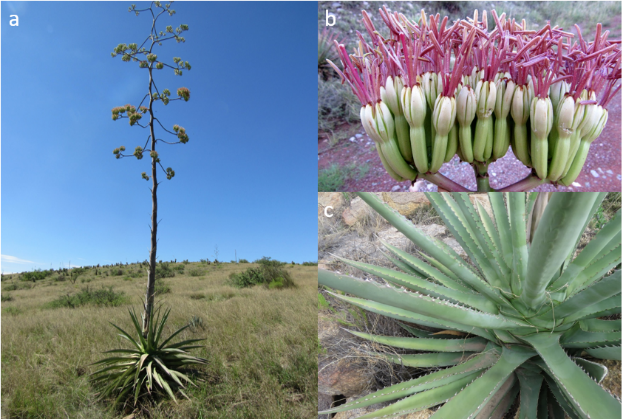


**Figure S 2:** *Agave palmeri* is a wild species presumably used for multiple purposes by pre- and post- contact cultures that grows ca 25 km from the extensive Hohokam agave fields near Marana, **a.** habit, often with tall, deep paniculate inflorescence with widely spaced lateral branches; **b.** flowers, characteristically thick with cream-maroon tepals and filaments; **c.** rosette and leaves**.**
