# Supplementary material for: Pre-contact Agave domesticates – living legacy plants in Arizona’s landscape
Source: Ann Bot. 2023 Oct 10;132(4):835–53. doi: 10.1093/aob/mcad113 (PMC10799993; doi:10.1093/aob/mcad113)
Supplement: mcad113_suppl_Supplementary_Figure_S3 [file mcad113_suppl_supplementary_figure_s3.docx]

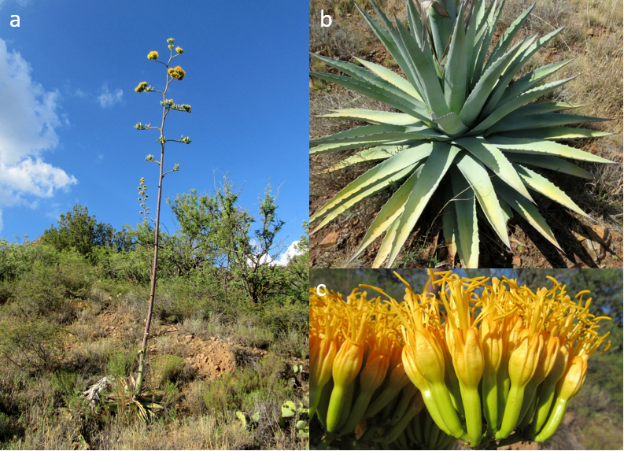


**Figure S 3:** *Agave chrysantha*, a wild species presumably used by pre- and post- cultures in Arizona, grows ca 28 km from the extensive Hohokam agave fields near Marana; **a.** habit, with tall, narrowly paniculate inflorescence; **b.** rosette and leaves, the latter variable with respect to color, shape, size and teeth; **c.** flowers, characteristically thickish, with tepals golden-yellow but sometimes cream to light yellow.
