# Supplementary material for: Pre-contact Agave domesticates – living legacy plants in Arizona’s landscape
Source: Ann Bot. 2023 Oct 10;132(4):835–53. doi: 10.1093/aob/mcad113 (PMC10799993; doi:10.1093/aob/mcad113)
Supplement: mcad113_suppl_Supplementary_Figure_S4 [file mcad113_suppl_supplementary_figure_s4.docx]

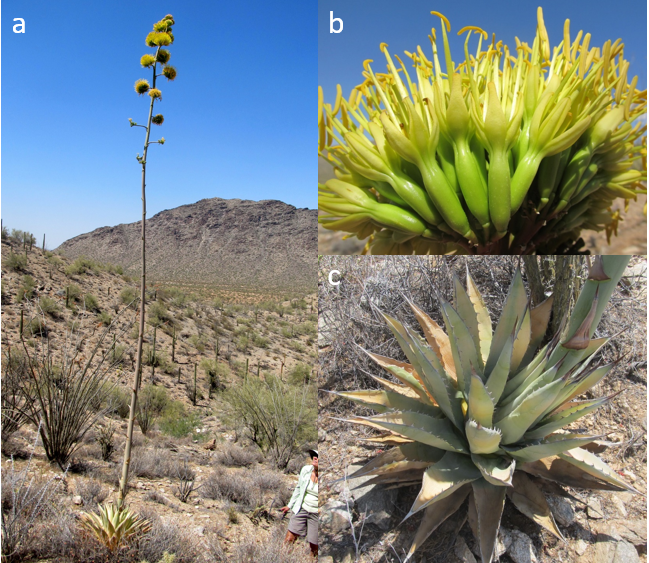


**Figure S 4:** *Agave simplex*, a wild species presumably used for multiple purposes by pre- and post- contact cultures, grows ca 35 km from the extensive Hohokam agave fields near Marana, **a.** habit, with tall, narrow paniculate inflorescence with short lateral branches; **b.** flowers, with tepals spreading, the tube shallow, light yellow; **c.** rosette and leaves, the latter typically broadly lanceolate, light green-glaucous, with numerous teeth.
