# Supplementary material for: Pre-contact Agave domesticates – living legacy plants in Arizona’s landscape
Source: Ann Bot. 2023 Oct 10;132(4):835–53. doi: 10.1093/aob/mcad113 (PMC10799993; doi:10.1093/aob/mcad113)
Supplement: mcad113_suppl_Supplementary_Figure_S5 [file mcad113_suppl_supplementary_figure_s5.docx]

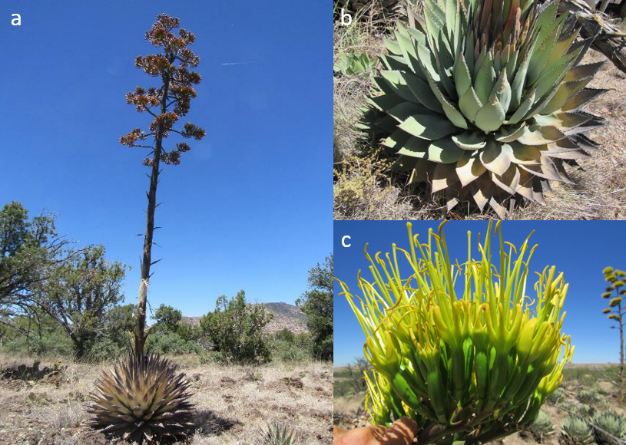


**Figure S 5:** *Agave parryi* var. *parryi*, a wild species used for multiple purposes by pre- and post- contact cultures, grows ca 30 km from the extensive Hohokam agave fields near Marana. Evidence suggests that var. *parryi*^1^ and var. *huachucensis*^2^ were cultivated by pre- and possibly post-contact^3^ peoples; a population occurs northwest of Sedona adjacent to a major archaeological habitation site where it grows with *A. verdensis, A. phillipsiana* and an unidentified agave; **a**. habit showing tightly clustered leaves and broad paniculate inflorescence; **b.** rosette with densely imbricate leaves; **c.** tall paniculate inflorescence with short lateral branches bearing yellow flowers that soon wilt.

**References**

1. Plogg, S. & Minnis, P. A study of the site specific distribution of *Agave parryi* in east central Arizona. *Kiva* **41,** 299-308 (1976) doi: [10.1080/00231940.1976.11757854](https://doi.org/10.1080/00231940.1976.11757854)

2. Parker, K., Trapnell, D., Hamrick, J. & Hodgson, W. Genetic and morphological contrasts between wild and anthropogenic populations of *Agave parryi* var. *huachucensis* in southeastern Arizona. *Annals of Botany* (2014) doi:10.1093/aob/mcu016, available online at [www.aob.oxfordjournals.org](http://www.aob.oxfordjournals.org).

3. Nabhan, G., Olmedo, J. & Matthew Pailes, M. The Huachuca Agave as "Mescal de la Mancha": translocation without domestication of *Agave parryi* var. *huachucensis* in the Sky Islands of the US and Mexico. *Desert Plants* **35**, 25-42 (2019).
