# Supplementary material for: Pre-contact Agave domesticates – living legacy plants in Arizona’s landscape
Source: Ann Bot. 2023 Oct 10;132(4):835–53. doi: 10.1093/aob/mcad113 (PMC10799993; doi:10.1093/aob/mcad113)
Supplement: mcad113_suppl_Supplementary_Figure_S6 [file mcad113_suppl_supplementary_figure_s6.docx]

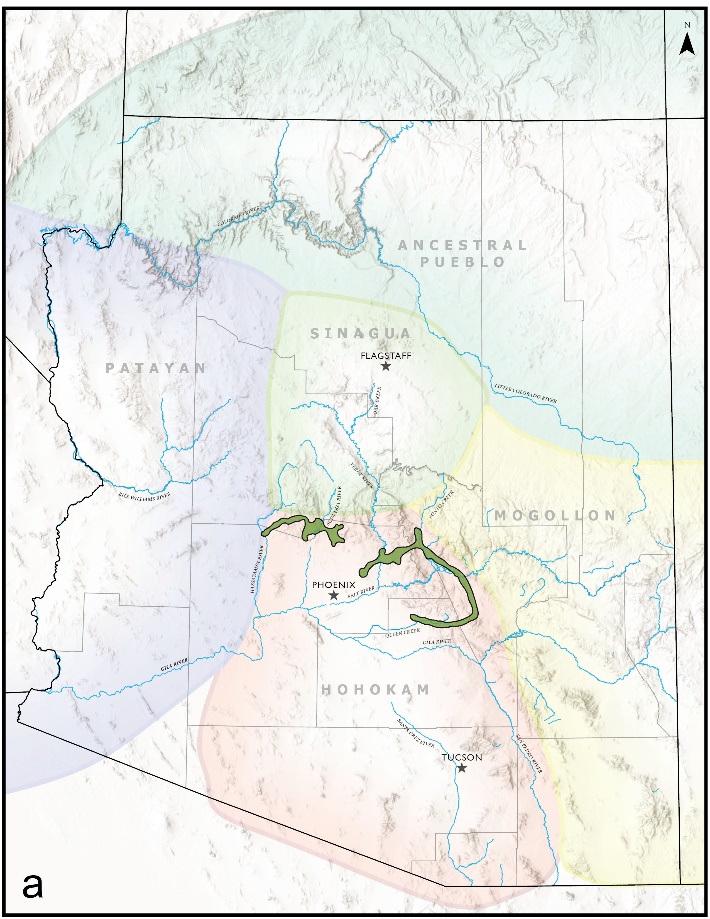

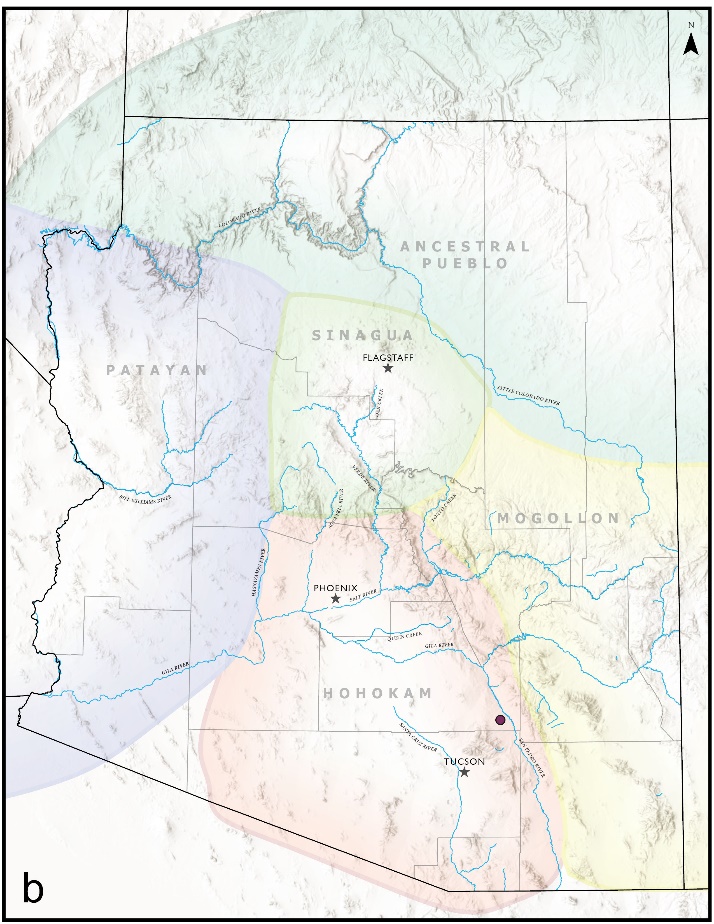


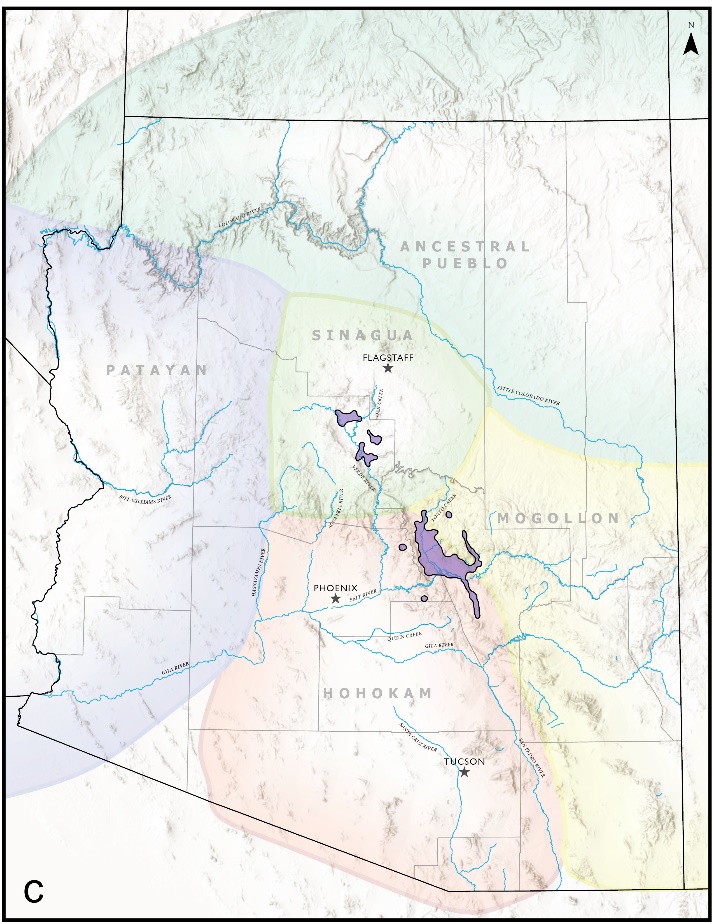

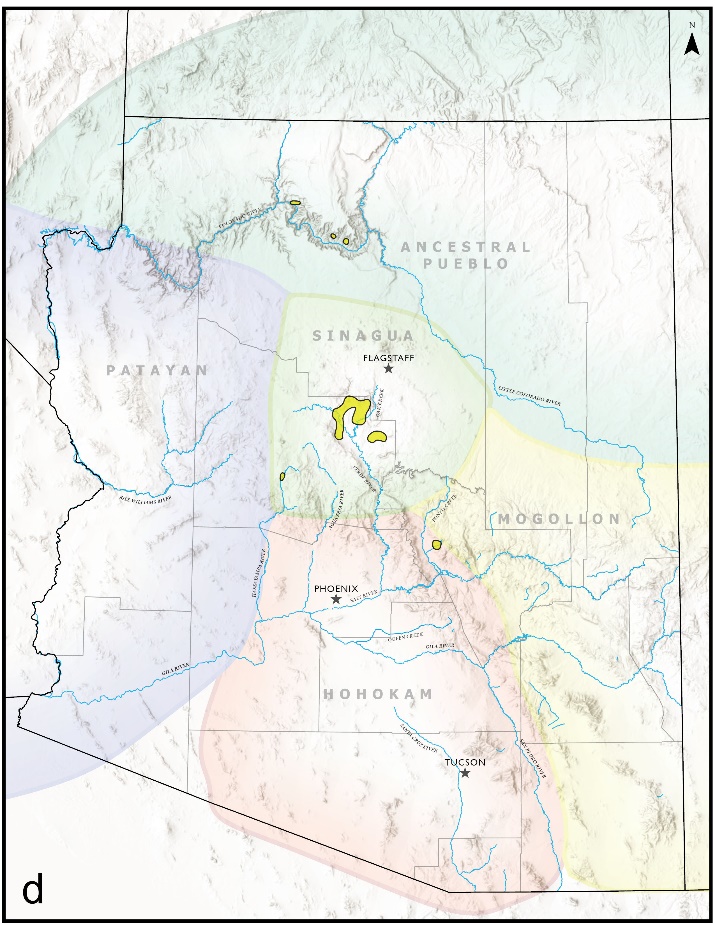


**Figure S 6**: Approximate areas of the PCADs and the cultures who farmed them prior to the 1350s. a. *Agave murpheyi*; b. *A. sanpedroensis*; c. *A. delamateri*; d. *A. phillipsiana.*
