# Supplementary material for: Pre-contact Agave domesticates – living legacy plants in Arizona’s landscape
Source: Ann Bot. 2023 Oct 10;132(4):835–53. doi: 10.1093/aob/mcad113 (PMC10799993; doi:10.1093/aob/mcad113)
Supplement: mcad113_suppl_Supplementary_Table_S1 [file mcad113_suppl_supplementary_table_s1.docx]

| Herbarium accession no. |  |  |
| --- | --- | --- |
| DES00008368 | DES00033685 | DES00051521 |
| DES00011695 | DES00034132 | DES00055125 |
| DES00012083 | DES00034133 | DES00064323 |
| DES00026929 | DES00034134 | DES00072674 |
| DES00026930 | DES00034135 | DES00077276 |
| DES00030426 | DES00034136 | DES00080234 |
| DES00030428 | DES00034167 | DES00080969 |
| DES00030805 | DES00034944 | DES00085780 |
| DES00031219 | DES00034945 | DES00085793 |
| DES00031225 | DES00035870 | DES00086026 |
| DES00032211 | DES00036297 | DES00086030 |
| DES00032682 | DES00036298 | DES00093617 |
| DES00032683 | DES00038129 | DES00093618 |
| DES00032684 | DES00043594 | DES00093645 |
| DES00032685 | DES00047154 | DES00093646 |
| DES00032686 | DES00050187 | DES00093821 |
| DES00032687 | DES00051399 | DES00093834 |
| DES00032688 | DES00051508 | DES00093835 |
| DES00032689 | DES00051509 | DES00093836 |
| DES00032690 | DES00051517 | DES00093965 |
| DES00032691 | DES00051518 | DES00093978 |
| DES00032692 | DES00051519 | DES00093979 |
| DES00032959 | DES00051520 |  |

**Table S 1.** Vouchered populations of *Agave murpheyi* deposited at Desert Botanical Garden herbarium; also available to view at <http://swbiodiversity.org/seinet/index.php>
