# Supplementary material for: Pre-contact Agave domesticates – living legacy plants in Arizona’s landscape
Source: Ann Bot. 2023 Oct 10;132(4):835–53. doi: 10.1093/aob/mcad113 (PMC10799993; doi:10.1093/aob/mcad113)
Supplement: mcad113_suppl_Supplementary_Table_S2 [file mcad113_suppl_supplementary_table_s2.docx]

| Herbarium accession |
| --- |
| no. |
| DES00032663 |
| DES00032664 |
| DES00032665 |
| DES00032666 |
| DES00032667 |
| DES00032668 |
| DES00032669 |
| DES00032670 |
| DES00032671 |
| DES00032672 |
| DES00032673 |
| DES00032674 |
| DES00032675 |
| DES00032676 |
| DES00032677 |
| DES00032678 |
| DES00032679 |
| DES00032680 |
| DES00032681 |
| DES00032807 |
| DES00032818 |
| DES00032819 |
| DES00032820 |
| DES00033675 |
| DES00033676 |
| DES00033677 |
| DES00033678 |
| DES00033679 |
| DES00033680 |
| DES00033681 |
| DES00033682 |
| DES00033683 |
| DES00033684 |
| DES00033688 |
| DES00033689 |
| DES00033690 |
| DES00033691 |
| DES00033692 |
| DES00033693 |
| DES00033694 |
| DES00033695 |
| DES00033697 |
| DES00034131 |
| DES00034146 |
| DES00034147 |
| DES00034148 |
| DES00034149 |
| DES00034150 |
| DES00034151 |
| DES00034152 |
| DES00034153 |
| DES00034154 |
| DES00034155 |
| DES00035120 |
| DES00035367 |
| DES00035371 |
| DES00035376 |
| DES00036295 |
| DES00036786 |
| DES00037971 |
| DES00039631 |
| DES00040055 |
| DES00050645 |
| DES00050653 |
| DES00051072 |
| DES00051073 |
| DES00051075 |
| DES00051076 |
| DES00051195 |
| DES00051548 |
| DES00052578 |
| DES00052580 |
| DES00053376 |
| DES00056052 |
| DES00056634 |
| DES00056639 |
| DES00056647 |
| DES00056650 |
| DES00056656 |
| DES00056657 |
| DES00056659 |
| DES00058312 |
| DES00058313 |
| DES00058618 |
| DES00059959 |
| DES00059960 |
| DES00060715 |
| DES00061486 |
| DES00061490 |
| DES00064101 |
| DES00064102 |
| DES00064174 |
| DES00064175 |
| DES00064176 |
| DES00064177 |
| DES00064178 |
| DES00064179 |
| DES00064180 |
| DES00064181 |
| DES00064187 |
| DES00064188 |
| DES00064246 |
| DES00064248 |
| DES00064278 |
| DES00068458 |
| DES00068459 |
| DES00070674 |
| DES00074147 |
| DES00074323 |
| DES00074324 |
| DES00074325 |
| DES00077119 |
| DES00077120 |
| DES00077278 |
| DES00077279 |
| DES00077280 |
| DES00078748 |
| DES00078749 |
| DES00078753 |
| DES00078754 |
| DES00078756 |
| DES00079064 |
| DES00079065 |
| DES00079066 |
| DES00079820 |
| DES00079821 |
| DES00079822 |
| DES00079823 |
| DES00079824 |
| DES00080116 |
| DES00080117 |
| DES00080121 |
| DES00080122 |
| DES00080123 |
| DES00080124 |
| DES00080125 |
| DES00080126 |
| DES00080127 |
| DES00080128 |
| DES00080129 |
| DES00080130 |
| DES00080136 |
| DES00080137 |
| DES00080138 |
| DES00080139 |
| DES00080140 |
| DES00080141 |
| DES00080151 |
| DES00080166 |
| DES00080167 |
| DES00080169 |
| DES00080170 |
| DES00080239 |
| DES00080240 |
| DES00080241 |
| DES00080242 |
| DES00080250 |
| DES00080436 |
| DES00080452 |
| DES00080453 |
| DES00080492 |
| DES00080493 |
| DES00080494 |
| DES00080700 |
| DES00080965 |
| DES00080968 |
| DES00080973 |
| DES00080975 |
| DES00081226 |
| DES00081227 |
| DES00081695 |
| DES00083129 |
| DES00083130 |
| DES00083956 |
| DES00083957 |
| DES00083961 |
| DES00083962 |
| DES00083973 |
| DES00083974 |
| DES00083975 |
| DES00084679 |
| DES00084680 |
| DES00084686 |
| DES00084687 |
| DES00084688 |
| DES00084691 |
| DES00084692 |
| DES00084693 |
| DES00085056 |
| DES00085057 |
| DES00085491 |
| DES00085492 |
| DES00085511 |
| DES00085514 |
| DES00085776 |
| DES00085792 |
| DES00085794 |
| DES00085795 |
| DES00085796 |
| DES00085799 |
| DES00085800 |
| DES00085821 |
| DES00085822 |
| DES00087082 |
| DES00087090 |
| DES00087091 |
| DES00087092 |
| DES00087094 |
| DES00087095 |
| DES00087101 |
| DES00087193 |
| DES00087194 |
| DES00087195 |
| DES00087198 |
| DES00089641 |
| DES00093560 |
| DES00093561 |
| DES00093604 |
| DES00093605 |
| DES00093857 |
| DES00093858 |
| DES00093861 |
| DES00093885 |
| DES00093906 |
| DES00093907 |
| DES00093909 |
| DES00093911 |
| DES00093912 |
| DES00093913 |
| DES00093914 |
| DES00093915 |
| DES00093916 |
| DES00093917 |
| DES00093919 |
| DES00093920 |
| DES00093966 |
| DES00093970 |
| DES00094128 |
| DES00094129 |
| DES00094130 |
| DES00094131 |
| DES00094132 |
| DES00094133 |
| DES00094134 |

**Table S 2.** Vouchered populations of *Agave delamateri* deposited at Desert Botanical Garden herbarium; also available to view at <http://swbiodiversity.org/seinet/index.php>
