# Supplementary material for: Pre-contact Agave domesticates – living legacy plants in Arizona’s landscape
Source: Ann Bot. 2023 Oct 10;132(4):835–53. doi: 10.1093/aob/mcad113 (PMC10799993; doi:10.1093/aob/mcad113)
Supplement: mcad113_suppl_Supplementary_Table_S3 [file mcad113_suppl_supplementary_table_s3.docx]

| Herbarium accession |
| --- |
| no. |
| DES00051077 |
| DES00051007 |
| DES00051006 |
| DES00051029 |
| DES00051028 |
| DES00051390 |
| DES00051391 |
| DES00051392 |
| DES00051394 |
| DES00051393 |
| DES00051512 |
| DES00051513 |
| DES00051514 |
| DES00051515 |
| DES00051516 |
| DES00051510 |
| DES00051511 |
| DES00051547 |
| DES00051712 |
| DES00052574 |
| DES00052573 |
| DES00056645 |
| DES00056633 |
| DES00058309 |
| DES00058966 |
| DES00061488 |
| DES00060714 |
| DES00065854 |
| DES00043023 |
| DES00040477 |
| DES00060713 |
| DES00068273 |
| DES00070706 |
| DES00038532 |
| DES00044332 |
| DES00044333 |
| DES00044334 |
| DES00068163 |
| DES00068164 |
| DES00068165 |
| DES00064434 |
| DES00064435 |
| DES00064436 |
| DES00064437 |
| DES00064438 |
| DES00064440 |
| DES00064441 |
| DES00064453 |
| DES00064454 |
| DES00064455 |
| DES00064457 |
| DES00068453 |
| DES00068455 |
| DES00068467 |
| DES00068468 |
| DES00068469 |
| DES00068470 |
| DES00080064 |
| DES00071006 |
| DES00071007 |
| DES00070707 |
| DES00071005 |
| DES00077122 |
| DES00077121 |
| DES00077124 |
| DES00077125 |
| DES00078740 |
| DES00078727 |
| DES00078747 |
| DES00078734 |
| DES00078744 |
| DES00078757 |
| DES00078794 |
| DES00078800 |
| DES00078745 |
| DES00078728 |
| DES00078741 |
| DES00080478 |
| DES00079005 |
| DES00079006 |
| DES00079007 |
| DES00080711 |
| DES00080713 |
| DES00080712 |
| DES00080715 |
| DES00081109 |
| DES00080071 |
| DES00080072 |
| DES00082709 |
| DES00082711 |
| DES00082714 |
| DES00082713 |
| DES00082692 |
| DES00084425 |
| DES00084444 |
| DES00084433 |
| DES00084462 |
| DES00084427 |
| DES00082693 |
| DES00082712 |
| DES00082710 |
| DES00084428 |
| DES00084429 |
| DES00084430 |
| DES00084431 |
| DES00084434 |
| DES00084435 |
| DES00084436 |
| DES00084437 |
| DES00084445 |
| DES00084446 |
| DES00084447 |
| DES00084463 |
| DES00084464 |
| DES00084465 |
| DES00084466 |
| DES00085463 |
| DES00086032 |
| DES00085464 |
| DES00085465 |
| DES00085466 |
| DES00093616 |
| DES00093564 |
| DES00093559 |
| DES00093558 |
| DES00093562 |
| DES00093563 |
| DES00093918 |
| DES00093921 |
| DES00093614 |
| DES00093615 |
| DES00093922 |
| DES00093923 |

**Table S 3.** Vouchered populations of *Agave philllipsiana* deposited at Desert Botanical Garden herbarium; also available to view at <http://swbiodiversity.org/seinet/index.php>
