# Supplementary material for: Pre-contact Agave domesticates – living legacy plants in Arizona’s landscape
Source: Ann Bot. 2023 Oct 10;132(4):835–53. doi: 10.1093/aob/mcad113 (PMC10799993; doi:10.1093/aob/mcad113)
Supplement: mcad113_suppl_Supplementary_Table_S4 [file mcad113_suppl_supplementary_table_s4.docx]

| Herbarium accession |
| --- |
| no. |
| DES00078831 |
| DES00079050 |
| DES00079049 |
| DES00079969 |
| DES00079046 |
| DES00079048 |
| DES00079043 |
| DES00079045 |
| DES00079051 |
| DES00079047 |
| DES00079044 |
| DES00078832 |
| DES00078833 |
| DES00079970 |
| DES00079971 |
| DES00079972 |
| DES00082046 |
| DES00082055 |
| DES00082047 |
| DES00082056 |
| DES00093403 |
| DES00093402 |
| DES00094650 |
| DES00094646 |
| DES00094647 |
| DES00094648 |
| DES00094651 |
| DES00094652 |

**Table S 4.** Vouchered populations of *Agave sanpedroensis* deposited at Desert Botanical Garden herbarium; also available to view at <http://swbiodiversity.org/seinet/index.php>
