# Supplementary material for: Pre-contact Agave domesticates – living legacy plants in Arizona’s landscape
Source: Ann Bot. 2023 Oct 10;132(4):835–53. doi: 10.1093/aob/mcad113 (PMC10799993; doi:10.1093/aob/mcad113)
Supplement: mcad113_suppl_Supplementary_Table_S5 [file mcad113_suppl_supplementary_table_s5.docx]

Herbarium accession

no.

| DES00078766 |
| --- |
| DES00078767 |
| DES00078793 |
| DES00078762 |
| DES00078763 |
| DES00078857 |
| DES00078858 |
| DES00078865 |
| DES00079153 |
| DES00079160 |
| DES00079155 |
| DES00079156 |
| DES00086637 |
| DES00086616 |
| DES00086613 |
| DES00086610 |
| DES00080093 |
| DES00080094 |
| DES00081229 |
| DES00081252 |
| DES00081235 |
| DES00080964 |
| DES00080966 |
| DES00080970 |
| DES00080972 |
| DES00080974 |
| DES00080977 |
| DES00080967 |
| DES00081230 |
| DES00081231 |
| DES00081232 |
| DES00081253 |
| DES00081254 |
| DES00081255 |
| DES00081263 |
| DES00083779 |
| DES00084137 |
| DES00084221 |
| DES00083157 |
| DES00084752 |
| DES00082640 |
| DES00082641 |
| DES00082694 |
| DES00082718 |
| DES00083217 |
| DES00084922 |
| DES00084907 |
| DES00084931 |
| DES00084924 |
| DES00084937 |
| DES00085887 |
| DES00085882 |
| DES00085528 |
| DES00085522 |
| DES00083780 |
| DES00084222 |
| DES00084223 |
| DES00084551 |
| DES00084753 |
| DES00084754 |
| DES00084925 |
| DES00084908 |
| DES00084909 |
| DES00084923 |
| DES00084932 |
| DES00084933 |
| DES00084934 |
| DES00084938 |
| DES00084939 |
| DES00084940 |
| DES00085569 |
|  |
| DES00085567 |
| DES00085581 |
| DES00085574 |
| DES00085575 |
| DES00085758 |
| DES00085741 |
| DES00085755 |
| DES00085529 |
| DES00085883 |
| DES00085884 |
| DES00085888 |
| DES00086614 |
| DES00086617 |
| DES00086638 |
| DES00089345 |
| DES00089340 |
| DES00089341 |
| DES00089342 |
| DES00089346 |
| DES00094573 |
| DES00093854 |
| DES00093548 |
| DES00093844 |
| DES00093840 |
| DES00093549 |
| DES00093699 |
| DES00093700 |
| DES00093859 |
| DES00093852 |
| DES00093860 |
| DES00093841 |
| DES00093842 |
| DES00093855 |
| DES00093853 |
| DES00093843 |
| DES00094574 |

**Table S 5.** Vouchered populations of *Agave verdensis* deposited at Desert Botanical Garden herbarium; also available to view at <http://swbiodiversity.org/seinet/index.php>
