# Supplementary material for: Pre-contact Agave domesticates – living legacy plants in Arizona’s landscape
Source: Ann Bot. 2023 Oct 10;132(4):835–53. doi: 10.1093/aob/mcad113 (PMC10799993; doi:10.1093/aob/mcad113)
Supplement: mcad113_suppl_Supplementary_Table_S6 [file mcad113_suppl_supplementary_table_s6.docx]

| Herbarium accession |
| --- |
| no. |
| DES00050641 |
| DES00050642 |
| DES00050643 |
| DES00050651 |
| DES00050652 |
| DES00051070 |
| DES00080817 |
| DES00080814 |
| DES00056653 |
| DES00061491 |
| DES00067132 |
| DES00067131 |
| DES00067130 |
| DES00067128 |
| DES00067129 |
| DES00064097 |
| DES00064099 |
| DES00087086 |
| DES00087084 |
| DES00087080 |
| DES00078773 |
| DES00078769 |
| DES00078746 |
| DES00078770 |
| DES00078771 |
| DES00078772 |
| DES00078774 |
| DES00078775 |
| DES00078776 |
| DES00079059 |
| DES00079060 |
| DES00079067 |
| DES00079061 |
| DES00079062 |
| DES00079063 |
| DES00079068 |
| DES00079069 |
| DES00080714 |
| DES00086083 |
| DES00086607 |
| DES00085069 |
| DES00080082 |
| DES00080980 |
| DES00080815 |
| DES00080890 |
| DES00080818 |
| DES00080819 |
| DES00080820 |
| DES00080898 |
| DES00080943 |
| DES00080971 |
| DES00084248 |
| DES00084227 |
| DES00084217 |
| DES00083968 |
| DES00082625 |
| DES00082626 |
| DES00084894 |
| DES00085524 |
| DES00083969 |
| DES00083970 |
| DES00084218 |
| DES00085860 |
| DES00085836 |
| DES00085841 |
| DES00085196 |
| DES00085445 |
| DES00085073 |
| DES00084540 |
| DES00084541 |
| DES00084542 |
| DES00084249 |
| DES00084250 |
| DES00084895 |
| DES00085197 |
| DES00085198 |
| DES00085199 |
| DES00085074 |
| DES00085075 |
| DES00085076 |
| DES00085070 |
| DES00085446 |
| DES00085447 |
| DES00085448 |
| DES00085525 |
| DES00085842 |
| DES00085843 |
| DES00085861 |
| DES00085862 |
| DES00086084 |

**Table S 6.** Vouchered populations of *Agave yavapaiensis* deposited at Desert Botanical Garden herbarium; also available to view at <http://swbiodiversity.org/seinet/index.php>
