# Supplementary material for: Pre-contact Agave domesticates – living legacy plants in Arizona’s landscape
Source: Ann Bot. 2023 Oct 10;132(4):835–53. doi: 10.1093/aob/mcad113 (PMC10799993; doi:10.1093/aob/mcad113)
Supplement: mcad113_suppl_Supplementary_Table_S7 [file mcad113_suppl_supplementary_table_s7.docx]

| **Species** |  | murpheyi | sanpedroensis | delamateri | phillipsiana | verdensis | yavapaiensis |
| --- | --- | --- | --- | --- | --- | --- | --- |
| Rosette height (m) |  | 0.6-1.2 | 0.5-0.7 | 0.6-1 | 0.75-1 | 0.5-0.6 | 0.5-0.6 |
| Heart size* |  | Medium | Medium | Medium | Medium | Medium | Large |
| Leaves |  |  |  |  |  |  |  |
|  | Leaf length (cm)* | 50-80 | 44-59 | 50-73 | 76-78 | 28-47 | 33-50 |
|  | Easily cut* | Yes | Yes | Yes | Yes | Yes | Yes |
|  | Teeth size* | Small | Small | Small | Vary | Small | Small |
|  | Teeth deflexed or straight* | Yes | Yes | Yes | Vary | Yes | Yes |
| Reproduction |  |  |  |  |  |  |  |
|  | Sexual | Rarely | No | No | No | Rarely | Rarely |
|  | Asexual |  |  |  |  |  |  |
|  | Bulbils* | Yes | No | No | No | No | No |
|  | Pups* | Yes | Yes | Yes | Yes | Yes | Yes |
| Flowering |  |  |  |  |  |  |  |
|  | Time* | Mar-Jun; Aug-Sept | Jul-Aug | Jun-Aug | (May-) Jun-Sept | Jun-Jul | Jun-Jul |
|  | Synchronous* | Yes | Yes | Yes | Yes | Yes | Yes |
| Fruit |  | Rarely | No | No | No | Few | Few |
| Seed |  | Rarely | No | No | No | Few | Few |
| Nativity |  | nw Sonora? | s AZ? | s AZ? | s AZ? | c AZ? | c AZ? |
| Chromosome No. |  | 2n^2^ | polyploid | 4n^3^ | 4n^3^ | 2n^4^ | 2n |
| Pollen viability |  | 20^2^,59, 92% | ? | (14)41-86% | 50-72% | (26)52-85% | 81-96% |
| Taste* (1-5 with 5 being very sweet) |  | 4 | ? | 5 | 5 | 5 | 5 |

**Table S 7a.**

| **Species** |  | simplex | chrysantha | palmeri | parryi | utahensis  kaibabensis |
| --- | --- | --- | --- | --- | --- | --- |
| Rosette height (m) |  | 0.3-0.7 | 0.5-1.2 | 0.4-1.3 | 0.15-0.7 | 0.15-0.9 |
| Heart size* |  | Small | Vary | Vary | Vary | Vary |
| Leaves |  |  |  |  |  |  |
|  | Leaf length (cm)* | 20-60 | 40-82 | 35-92 | 7-65 | 25-50 |
|  | Easily cut* | No | No | Vary | No | No |
|  | Teeth size* | Small | Vary | Small | Vary | Small |
|  | Teeth deflexed or straight* | Vary | Vary | Yes | Yes | Vary |
| Reproduction |  |  |  |  |  |  |
|  | Sexual | Yes | Yes | Yes | Yes | Yes |
|  | Asexual |  |  |  |  |  |
|  | Bulbils* | No | No | No | No | No |
|  | Pups* | Rarely | Rarely | Rarely | Yes | No |
| Flowering |  |  |  |  |  |  |
|  | Time* | Jun-Jul | Jun-Aug | Jul-Sep | May-Jun | May-Jun |
|  | Synchronous* | No | No | No | Vary | No |
| Fruit |  | Yes | Yes | Yes | Yes | Yes |
| Seed |  | Much | Much | Much | Much | Much |
| Nativity |  | s NV, AZ | AZ | AZ, NM, n Sonora | AZ, NM, n Mexico | AZ |
| Chromosome No. |  | 2n^5^ | 2n^2,3^ | 2n^2,3,6^ | 4n^2,3^ | 2n^3,4^ |
| Pollen viability |  | ? | 98% | 83, 93%^2^ | ? | 87% |
| Taste* (1-5 with 5 being very sweet) |  | ? | 4 | ? | 4 | 4 |

**Table S 7b**

**Table S 7a, b.** Characteristics of Arizona cultivated agaves (a) and regional wild agaves (b; both adapted from Hodgson 2013^1^). Cloning helped fix desirable characteristics such as easily cut leaves, small, downturned teeth, production of bulbils and pups, synchrony and timing of flowering, and taste. Three of the six PCADs are polyploids; polyploidy can create a diversity of novel phenotypic traits for which people can select. Pollen viability of *Agave delamateri* plants ranged widely, from 14 and 22% in Tonto Basin to 41-86% in Verde Valley. The public compared the taste of regional wild and the PCADs baked at three agave roasts in 2007, 2008, and 2009. Tasters considered the domesticated species, especially *A. phillipsiana*, to be sweeter than the wild species. Distribution, reproductive and morphological characteristics, and flowering times are based on observations and specimens deposited at DES (see Table SI-1 – SI-6 for PCDAs), the specimens available to view at <http://swbiodiversity.org/seinet/index.php>. Additional data for chromosome number and pollen viability from Hodgson, W. *Investigations of four rare pre-Columbian cultivated agaves (Agavaceae) in central Arizona*. Final Report to US Fish & Wildlife Service (2007); and unpublished data on pollen viability analyses of agaves from Living Collection, Desert Botanical Garden (provenance data available at <https://livingcollections.org/dbg/Home.aspx>); copies deposited at Desert Botanical Garden, Phoenix.

* Character possibly selected for by farmers; bulbil production on undamaged flower stalk

**References**

1. Hodgson, W. in *Explorations in Ethnobiology: the legacy of Amadeo Rea* (eds Quinlan, M. & Lepofsky, D*.*) 78-103 (Society of Ethnobiology, Denton, Texas, 2013).

2. Pinkava, D. & Baker, M. Chromosome and Hybridization Studies of Agaves. Desert Plants, **7**, 93-100 (1985).

3. Reveal, J. & Hodgson, W. Agave. L. in: *Flora of North America* (ed Flora of North America Editorial Committee) **26,** 442-461 (Oxford University Press, Oxford, 2002).

4. Baker, M., Rebman, J., Parfitt, B., Pinkava, D., Christy, C., Salywon, A. & Puente-Martinez, R. chromosome numbers of miscellaneous angiosperm taxa. *Bot. Res. Inst. Texas* **3**, 279 – 283 (2009).

5. Pinkava, D.J., Baker, M., Johnson, Trushell, N., Ruffner, G., Felger, R. & Van Devender, R.

1992. Additions, Notes and Chromosome Numbers for the Flora of Vascular Plants of Organ Pipe Cactus National Monument, Arizona. *Journal of the Arizona-Nevada Academy of Science* **24-25,** 13-18 (1992).

6. Bennett, M. & Smith, J. Nuclear DNA amounts in angiosperms, *Philosophical Transactions of the Royal Society of London*, **334**, 309-345 (1991).
