# Supplementary material for: Pre-contact Agave domesticates – living legacy plants in Arizona’s landscape
Source: Ann Bot. 2023 Oct 10;132(4):835–53. doi: 10.1093/aob/mcad113 (PMC10799993; doi:10.1093/aob/mcad113)
Supplement: mcad113_suppl_Supplementary_Table_S9 [file mcad113_suppl_supplementary_table_s9.docx]

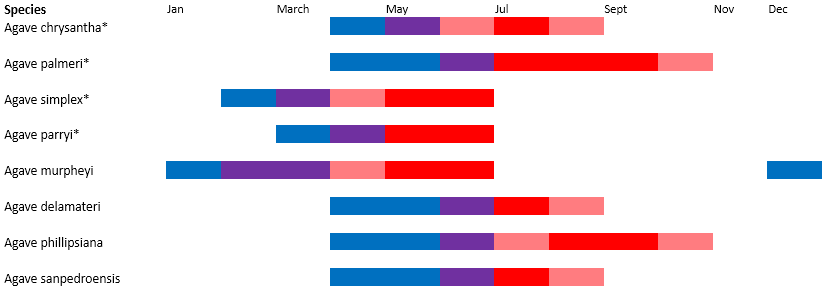


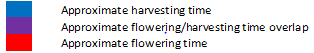


**Table S 9.** Approximate harvest time and flowering times of wild agaves (indicated with an asterisk) and the presumed domesticated species accessible to Hohokam (adapted from Hodgson 2013^1^). PCDAs exhibit synchrony in flowering, allowing easier harvesting of the “hearts” as these will be in the same stage of development, unlike the more variable, and generally longer flowering times of wild agaves. Planting different agaves having different flower periods extended the harvest period. Growing different kinds also promoted optimal resource production adapted to local environmental or ecological conditions and uses. We define the flowering period as the time the first lateral branches develop mature flowers to the end of flowering in the uppermost branches. Flowering times based on observations and herbarium specimens (see also Reveal & Hodgson 2001^2^), the specimens available to view at <http://swbiodiversity.org/seinet/index.php>.

**References**

1. Hodgson, W. in *Explorations in Ethnobiology: the legacy of Amadeo Rea* (eds Quinlan, M. & Lepofsky, D*.*) 78-103 (Society of Ethnobiology, Denton, Texas, 2013).

2. Reveal, J. & W. Hodgson. Agave. L. in: *Flora of North America* (ed Flora of North America Editorial Committee) **26,** 442-461 (Oxford University Press, Oxford, 2002).
